# STK3 is a transcriptional target of YAP1 and a hub component in the crosstalk between Hippo and Wnt signaling pathways during gastric carcinogenesis

**DOI:** 10.1186/s12943-025-02391-x

**Published:** 2025-07-02

**Authors:** Fuda Xie, Yang Lyu, Bonan Chen, Hoi Wing Leung, Peiyao Yu, Tiejun Feng, Canbin Fang, Alvin H.K. Cheung, Bin Zhou, Jianhui Jiang, Ge Zhang, Dazhi Xu, Liang Li, Chen Jiang, Jianwu Chen, Zhaocai Zhou, Liwei An, Bing Huang, Kangmin Zhuang, Xiaobei Luo, Kam Tong Leung, Ching Hei To, Brigette BY Ma, Chi Chun Wong, William KK Wu, Jun Yu, Ka Fai To, Wei Kang

**Affiliations:** 1 Department of Anatomical and Cellular Pathology, State Key Laboratory of Translational Oncology, Prince of Wales Hospital, Sir Y.K. Pao Cancer Center, The Chinese University of Hong Kong, Hong Kong, China; 2 Institute of Digestive Disease, State Key Laboratory of Digestive Disease, Li Ka Shing Institute of Health Science, The Chinese University of Hong Kong, Hong Kong, China; 3 CUHK-Shenzhen Research Institute, Shenzhen, China; 4 Department of Anesthesiology, Renmin Hospital, Wuhan University, Wuhan, China; 5 Guangdong Provincial Key Laboratory of Malignant Tumor Epigenetics and Gene Regulation, Medical Research Center, Sun Yat-Sen Memorial Hospital, Sun Yat-Sen University, Guangzhou, China; 6 Law Sau Fai Institute for Advancing Translational Medicine in Bone and Joint Diseases (TMBJ), School of Chinese Medicine, Hong Kong Baptist University, Hong Kong, China; 7 Department of Gastric Surgery, Department of Oncology, Shanghai Medical College, Fudan University Shanghai Cancer Center, Fudan University, Shanghai, China; 8 Department of Pathology, Guangdong Province Key Laboratory of Molecular Tumor Pathology, Nanfang Hospital and Basic Medical College, Southern Medical University, Guangzhou, China; 9 Department of Pathology, State Key Laboratory of Oncology in South China, Guangdong Provincial Clinical Research Center for Cancer, Sun Yat- Sen University Cancer Center, Guangzhou, China; 10 Department of Burn and Plastic Surgery, General Hospital of Southern Theater Command, PLA, Guangzhou, China; 11 State Key Laboratory of Genetic Engineering, School of Life Sciences, Zhongshan Hospital, Fudan University, Shanghai, China; 12 Department of Stomatology, Department of Biochemistry and Molecular Biology, Shanghai Tenth People’s Hospital, Tongji University School of Medicine, Shanghai, China; 13 Guangdong Provincial Key Laboratory of Gastroenterology, Department of Gastroenterology, Nanfang Hospital, Southern Medical University, Guangzhou, China; 14 Department of Pediatrics, The Chinese University of Hong Kong, Hong Kong, China; 15 Department of Clinical Oncology, Prince of Wales Hospital, The Chinese University of Hong Kong, Hong Kong, China; 16 Department of Anaesthesia and Intensive Care, The Chinese University of Hong Kong, Hong Kong, China; 17 Department of Medicine and Therapeutics, The Chinese University of Hong Kong, Hong Kong, China

**Keywords:** STK3, YAP1, Wnt signaling, Gastric cancer

## Abstract

**Background:**

Serine/threonine kinase 3 (STK3) is recognized as a key regulator in Hippo pathway and a tumor-suppressing gene in various cancer types. However, its non-canonical role has been gradually revealed in cancer development.

**Methods:**

Our objective is to elucidate the upregulation pattern and molecular mechanisms of STK3 in advancing gastric cancer (GC) progression. The regulation of YAP1 on STK3 was assessed through a combination of bulk and single-cell RNA-sequencing, Western blot, ChIP-qPCR, gene knockout mouse models, and functional rescue assays. The oncogenic roles of STK3 were confirmed through subcutaneous xenograft formation models and functional assays including spheroid formation and organoid growth. The phosphorylated target of STK3 was revealed by co-immunoprecipitation and *in vitro* kinase assays. STK3-targeted drugs were screened out by molecular docking and cellular thermal shift assay (CETSA).

**Results:**

Reduction of YAP1 significantly impaired STK3 expression at both mRNA and protein levels, and deletion of STK3 partially attenuated the oncogenic activity of YAP1. Notably, MNNG-induced tumors in *Yap1*^*−/−*^*Taz*^*−/−*^ mice exhibited decreased STK3 expression. Knockdown of STK3 led to reduced expression of stemness markers and xenograft growth, while sensitizing GC organoids and xenografts to 5-fluorouracil treatment. Mechanistically, the direct interaction between STK3 and GSK-3β promoted GSK-3β phosphorylation and β-catenin nuclear accumulation, and thus the activation of Wnt signaling. Furthermore, aminopterin demonstrates as a promising STK3-targeted small molecule with remarkable effectiveness in inhibiting GC cell malignance and xenograft growth.

**Conclusions:**

STK3 was identified as a transcriptional target of YAP1, leading to enhanced DNA repair ability and stemness acquisition during GC progression by activating Wnt/β-catenin activity through GSK-3β degradation. Moreover, STK3-targeted therapy offered a novel approach to concur acquired chemo-resistance in GC patients.

**Graphical Abstract:**

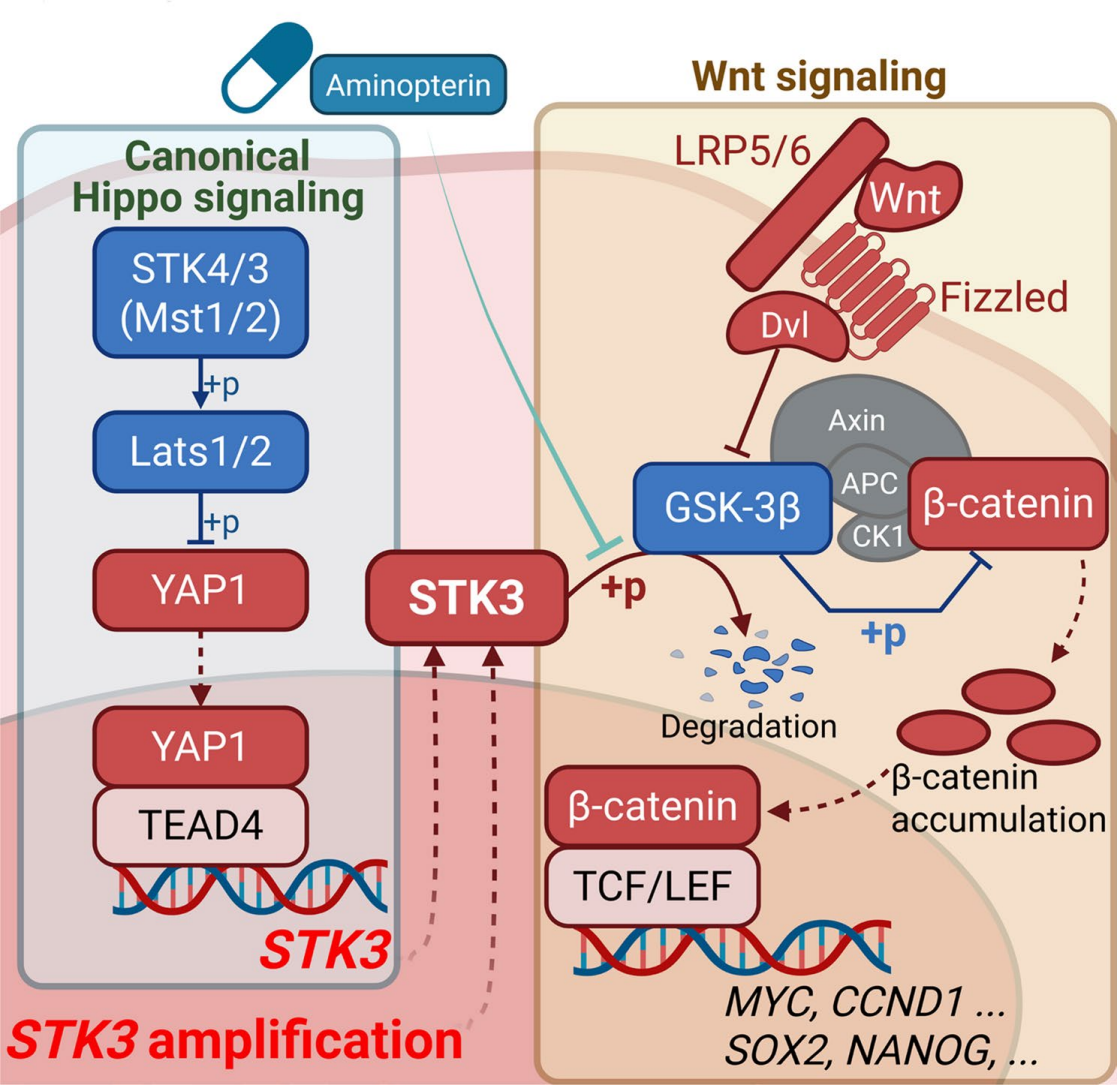

**Supplementary Information:**

The online version contains supplementary material available at 10.1186/s12943-025-02391-x.

## Introduction

Gastric cancer (GC) remains an increasingly severe world health issue worldwide and is more common in Asian countries. According to the Global Cancer Statistics 2022, GC was certified as the 5th most frequently occurring carcinogenesis and the 4th leading cause of cancer-related death all over the world [1]. Comprehension and treatment strategies for GC patients have undergone significant development over recent decades. Various clinical approaches have been employed for GC patients, showcasing notable clinical benefits while maintaining acceptable side effects [2, 3]. However, the emergence of drug resistance due to prolonged treatment remains a formidable challenge in GC therapy [4]. Therefore, there is still an urgent need to unravel the underlying molecular mechanisms to address the acquired resistance to conventional therapies effectively.

The Hippo pathway is commonly recognized as a significant participant in multiple cancer progression. Briefly, the serine/threonine-protein kinases 4/3 (STK4/3, also known as mammalian Ste20-like kinases MST1/2), along with salvador homolog 1 (SAV1), initiate the signaling transduction by phosphorylating the large tumor suppressor 1/2 kinases (LATS1/2) and subsequently the downstream transcriptional coactivator Yes-associated protein 1 (YAP1). Phosphorylated YAP1 will be sequestered in the cytoplasm and interact with 14-3-3 complex, leading to its degradation through a ubiquitination-dependent process [5]. In pathological conditions, the deactivation of the Hippo pathway will result in the de-phosphorylation and nuclear translocation of YAP1. Within the nucleus, YAP1 can engage with transcription factors to stimulate cell proliferation and suppress apoptosis, potentially causing organ enlargement and fostering tumorigenesis [6]. Mainly induced by *Helicobacter pylori* (*H. pylori*) infection, the hyperactivation of Hippo-YAP1 signaling is commonly observed in GC patients and demonstrates close correlation with aggressive characteristics of cancer cells [7–9]. Meanwhile, Our previous work demonstrated that the YAP1-mediated CTGF activation contributed to the resistance of chemotherapy in GC patients by enhancing microenvironment stiffness [10]. Therefore, there is noteworthy clinical potential in elucidating the transcriptional targets of YAP1 that could accelerate GC progression and therapy resistance, providing valuable insights for clinical implementation.

We commenced this study with an initial endeavor to identify novel downstream oncogenes of the YAP1 signaling by investigating the RNA sequencing (RNA-seq) results of siRNA-mediated YAP1 knockdown in GC cell lines. Surprisingly, STK3 emerged as the sole downregulated member among all serine/threonine kinases following YAP1 deletion. This unexpected discovery prompted further experiments to elucidate the potential bidirectional regulation between YAP1 and STK3. Upon deeper analysis of STK3 expression patterns in GC patients, we unveiled a frequent amplification of STK3 and significant correlation with poorer clinical outcomes, suggesting that this widely recognized tumor suppressor gene may exhibit oncogenic properties in GC progression.

Building upon these findings, our objective was to delve into the oncogenic roles of STK3 and the underlying molecular mechanisms. Through bioinformatic analysis and both *in vitro* and *in vivo* validations, we illustrated the pro-tumorigenic functions of STK3 in regulating DNA damage response and stemness acquisition. Mechanistically, STK3 was found to directly interact with GSK-3β, leading to its phosphorylation, which would abolish the nuclear translocation of β-catenin and subsequent activation of the Wnt signaling pathway. In addition, we performed virtual screening and *in vivo* validation of novel STK3 inhibitors, aiming to enrich our armamentarium against chemoresistance in GC treatment.

## Materials and methods

### Cell lines, organoids, primary samples, and clinical cohorts

Human GC cell lines MKN1, MKN7, MKN28, and NCI-N87 were purchased from American Type Culture Collection (ATCC) and routinely maintained in a humidified incubator at 37ºC in 5% CO_2_. The culture medium was composed of RPMI-1640 medium (Gibco), 10% fetal bovine serum (FBS, Gibco), and 1% penicillin-streptomycin (PS, Gibco). The conduction of GC organoid models was in accordance with previously instituted protocol [11] and as follows: GC tumor tissues, sizing from 0.5 to 1 cm^3^, were collected, rinsed, minced, and incubated at 37◦C for 1 h. The suspension was quenched using a cold culture medium, followed by filtration through a 70-micrometer strainer and then centrifugation at 400 g for 5 min. Cell pellets were resuspended with Matrigel (BD Biosciences) and seeded in the well to establish a 3D culture model. The Hong Kong GC tissue microarray cohort comprising 278 cases was collected between 2002 and 2014 at Prince of Wales Hospital.

### *In vitro* functional assays

Cell transfections in this work were carried out using Lipofectamine 2000 Transfection Reagent (ThermoFisher Scientific). The information of siRNAs and plasmids adopted in this study was recorded in Supplementary Table S1-2, and primary and secondary antibodies adopted in Western blot were recorded in Supplementary Table S3. Detailed procedures of functional assays, such as monolayer colony formation, cell invasion, and spheroid formation assays, were recorded in Supplementary Materials and Methods.

### Immunohistochemistry (IHC) staining

The information on antibodies adopted in IHC staining was provided in Supplementary Table S4. The procedure of IHC was as follows: after deparaffinization and dehydration, tissue sections in 5 μm were treated with ethylenediaminetetraacetic acid (EDTA) antigen retrieval solution (Beyotime) for antigen retrieval. The sections were then incubated with primary antibodies overnight at 4℃ and subsequently incubated with HPR-conjugated secondary antibodies. Treatment with DAB Substrate Kit (Dako) was performed to achieve visualization of protein expression. The immunoreactive score for protein expression was quantified based on the percentage of positively stained tumor cells and staining intensity, and analyses were conducted using ImageJ software and recorded in Supplementary Table S5.

### Fluorescence *in situ* hybridization (FISH)

STK3 probe (Empire Genomics) was employed to detect the copy number changes. PreTreatment Kit 1 (KA2375, Abnova) was performed to pretreat formalin-fixed, paraffin-embedded (FFPE) tissue sections. Copy number gain (CNG) (3–5 spots per cell) and gene amplification (copy number > 5 per cell) were defined depending on the probe signal.

### Immunofluorescence (IF) assay

Slides embedded with treated cells were initially fixed by 4% paraformaldehyde (PFA) and then blocked in a Phosphate buffer saline (PBS) buffer containing 0.1% Triton X-100 (Sigma-Aldrich) and 1% bovine serum albumin (BSA, Millipore). Subsequently, the slides were incubated overnight at 4℃ with primary antibodies, followed by a one-hour incubation at room temperature with corresponding secondary antibodies. Details of the primary and secondary antibodies are provided in Supplementary Table S6. For nuclear staining, the sections were treated with 1 µg/mL DAPI (Sigma-Aldrich) for 15 min at room temperature. Imaging was performed by adopting a Zeiss laser scanning microscopy (LSM) 880 confocal microscope.

### RNA extraction and RNA-seq analysis

The total RNA of YAP1 or STK3 knockdown cells were extracted by RNeasy kit (Qiagen). RNA quality was assessed by Tapestation (Agilent). The library was prepared using the Illumina Truseq RNA Kit (Illumina). The sequencing was conducted by the NovaSeq 6000 platform (Illumina) with single end read 100 bp. Reads were quality-checked with “FastQC” (v0.12.0), and “Trim_galore” (v0.6.4) was adopted for sequence trimming. The raw sequencing reads were aligned to the Homo sapiens genome assembly GRCh37 (hg19) from the NCBI database using “HISAT2” (v2.1.0) [12]. Gene expressions were quantified by “SAMtools” (v1.14) [13] and “FeatureCount” (v1.6.4) [14]. The differentially expressed genes (DEGs) were identified by the R package “DESeq2” (v1.38.1) [15]. The enrichment analysis based on Gene Ontology (GO) and Kyoto Encyclopedia of Genes and Genomes (KEGG) database was performed by the R package “ClusterProfiler” (v4.6.2) [16]. The gene set enrichment analysis (GSEA) quantifies the enrichment level through the running sum score beneath the enrichment curve, where the absolute value of enrichment score (ES) reflects the strength of enrichment, and the sign of ES reveals its direction of signaling regulation. The GSEA was conducted by R package “ClusterProfiler” (v4.6.2). We also uploaded the processed bulk-RNA-seq data generated in this work to Supplementary Table S7.

### Public dataset-based bioinformatic analysis

Two public GC cohorts were adopted in this work, namely The Cancer Genome Atlas-stomach adenocarcinoma cohort [17] (TCGA-STAD) and the Asian Cancer Research Group (ACRG) cohort [18]. For the functional enrichment analysis regarding the *STK3* expression level, we first evaluated the whole genomic expression level alteration by comparing the 10% samples (*n* = 37) with the lowest *STK3* expression (*STK3*^*−*^ samples) and the 10% samples with the highest *STK3* expression (*STK3*^*+*^ samples). DEGs were identified by R package “DESeq2” with the criteria of| log_2_(Fold Change)| > 1 and *P*-value < 0.05. The enrichment analysis were performed using R package “ClusterProfiler”. Pearson’s correlation analysis was conducted based on data from TCGA, ACRG, Cancer Cell Line Encyclopedia (CCLE, https://sites.broadinstitute.org/ccle/), and Clinical Proteomic Tumor Analysis Consortium (CPTAC, https://proteomics.cancer.gov/programs/cptac) (calculated by R package “stats” (v4.1.3)). The CCLE database includes the mRNA expression profile of 1450 cell lines belonging to multiple organs including stomach. The expression data was presented in log_2_(TPM + 1) and downloaded from online database DepMap portal (https://depmap.org/portal/data_page/) [19]. The binding motifs of YAP1/TEAD4 on the corresponding promoter region of STK3 were predicted by the Eukaryotic Promoter Database (https://epd.epfl.ch//index.php) and JASPAR 2022 database (https://jaspar.genereg.net). The drug sensitivity analysis was conducted by an online server Gene Set Cancer Analysis (https://guolab.wchscu.cn/GSCA/#/drug, GSCA). The IC50 of 265 small molecules in 860 cell lines and its corresponding mRNA gene expression profile from Genomics of Drug Sensitivity in Cancer (GDSC) were collected and merged by GSCA. Pearson correlation analysis was performed to get the correlation between *STK3* mRNA expression and drug IC50. *P*-value was adjusted by false discovery rate (FDR) [20]. The original data of chromatin immunoprecipitation sequencing (ChIP-seq) can be accessed in GEO database under the accession number GSE44416 [21]. The analysis and visualization were achieved by the online tool UCSC Genome Browser (https://genome.ucsc.edu/). Generally, the ChIP-seq data was retrieved from Cistrome Data Browser (http://cistrome.org/db/#/) and then imported into UCSC Genome Browser. Then the region of observation in the track illustrations was narrowed into STK3 encoding area (chr8:98433047–98825712) and then zoomed out by 1.5 times to include the promoter region. The illustration was downloaded through the “View– PDF” module.

### Single-cell RNA-seq (scRNA-seq) analysis

The scRNA-seq analysis was carried out based on a public dataset (https://dna-discovery.stanford.edu/research/datasets/, “Gastric scRNA-seq” dataset) [22] using the R package “Seurat” (v4.0.2) [23]. Cells expressing fewer than 200 genes, having more than 20% mitochondrial genes, or exhibiting an outlier number of unique molecular identifiers were removed. Genes detected in fewer than 3 cells were also excluded. Data normalization was conducted according to default settings [22]. The gene markers for cluster labeling and the reference for the selection of these markers were recorded in Supplementary Table S8. The “DimPlot”, “DotPlot” “FeaturePlot”, and “VlnPlot” functions integrated in “Seurat” package were adopted for visualization. The *STK3*^*+*^ cells were identified as cells with upregulated *STK3* expression level within the “cancer cell” cluster. The Gene Set Variation Analysis (GSVA) was conducted by R package “GSVA” (v1.46.0) [24]. The relative differentiate state of cells was predicted by R package CytoTRACE (v0.3.3) [25]. Additionally, single-cell pseudotime analysis was performed by Monocle2 in the R package “monocle” (v2.27.0) [26]. After filtering and dimension-reducing under the recommended parameters and procedure, the cells were ordered and visualized with the functions “plot_cell_trajectory” and “plot_pseudotime_heatmap”.

### Structure-based virtual screening

The 3D structure files of STK3 were retrieved from Protein Data Bank (PDB, https://www.rcsb.org/) with the accession number “8A66”. The candidate compound library was composed of 4511 small molecules with literature evidence proving their anti-cancer effectiveness. Molecular docking was performed by Autodock 4.2.6 [27], and Lamarckian genetic algorithm was applied for the docking procedure. The detailed parameters were recorded in Supplementary Table S9. Information of the compound library and their corresponding binding affinities with STK3 were recorded in Supplementary Table S10.

### *In vivo* studies

The *Yap1*^*floxed/+*^;*Taz*^*floxed/+*^ murine line was obtained by crossing *Yap1*^*floxed/+*^ mice (a kind gift from professor Duojia Pan, UT Southwestern Medical Center) and *Taz*^*floxed/+*^ mice (produced by Biocytogen, Beijing, China). Genomic DNA extracted from tail biopsies was used to evaluate offspring genotype. *Ubc-Cre/ERT2* mice were purchased from the Jackson laboratory. *Yap1*^*−/−*^*Taz*^*−/−*^ mice were generated by cross-breeding *Yap1*^*floxed/floxed*^; *Taz*^*floxed/floxed*^ with the tamoxifen-inducible Ubc-Cre/ERT2 mice [28]. For subcutaneous xenograft formation assays, NCI-N87 cells (10^6^ cells/mouse) were treated with shCtrl or shSTK3 (Vector backbone: pLKO.1; target sequence: 5’-CGGCGCCUAAGAGUAAACUAA-3’(shSTK3-1); 5’-GGACUACUUUGAUAAGCAA-3’ (shSTK3-2)) and then subcutaneously injected into 4-week-old NOD scid gamma (NSG) mice (*n* = 10/group). Tumor size was measured every two days by a digital caliper. The mice were sacrificed 16 days after the injection, and the xenografts were harvested, weighted, and possessed for further examination. For evaluating the synergistic effect of STK3 depletion and 5-fluorouracil (5-FU), four groups of NSG mice (*n* = 6/group) were subcutaneously inoculated with NCI-N87 cells and administered with vehicle (PBS), shSTK3, 5-FU (20 mg/kg, twice per week), shSTK3 + 5-FU, respectively. For evaluating the synergistic effect of aminopterin (Amin) and 5-FU, four groups of NSG mice (*n* = 6/group) were subcutaneously inoculated with NCI-N87 cells and administered with vehicle (PBS), Amin (0.15 mg/kg, twice per week), 5-FU (10 mg/kg, twice per week), Amin + 5-FU, respectively. The mice were sacrificed after 28-day administration, and the xenografts were harvested and evaluated. All mice experiments were authorized by the Animal Ethics Experimentation Committee (AEEC) from CUHK. The records for xenograft weights are recorded in Supplementary Table S11, and the parameters for power analysis are detailed in Supplementary Table S12.

### Statistical analysis

Two-tailed student’s t-test was adopted to demonstrate the significance between assay groups and control in gene expression level comparison and the functional assays. Pearson’s correlation was used to conduct correlation analysis. Statistical analyses were performed by Graph Pad Prism 8.0 (GraphPad). The co-expression index of *STK3* and *YAP1* was defined as the sum of their expression levels in the same sample. The clinical significance of gene expression was assessed by Kaplan-Meier curve and conducted by R package “survival”. Data were expressed as mean ± standard error of the mean (SEM) of triplicate independent experiments. Two-tailed *P*-value of less than 0.05 was defined as statistically significant, and *P*-value of less than 0.001 was considered highly significant.

## Results

**Fig. 1:**
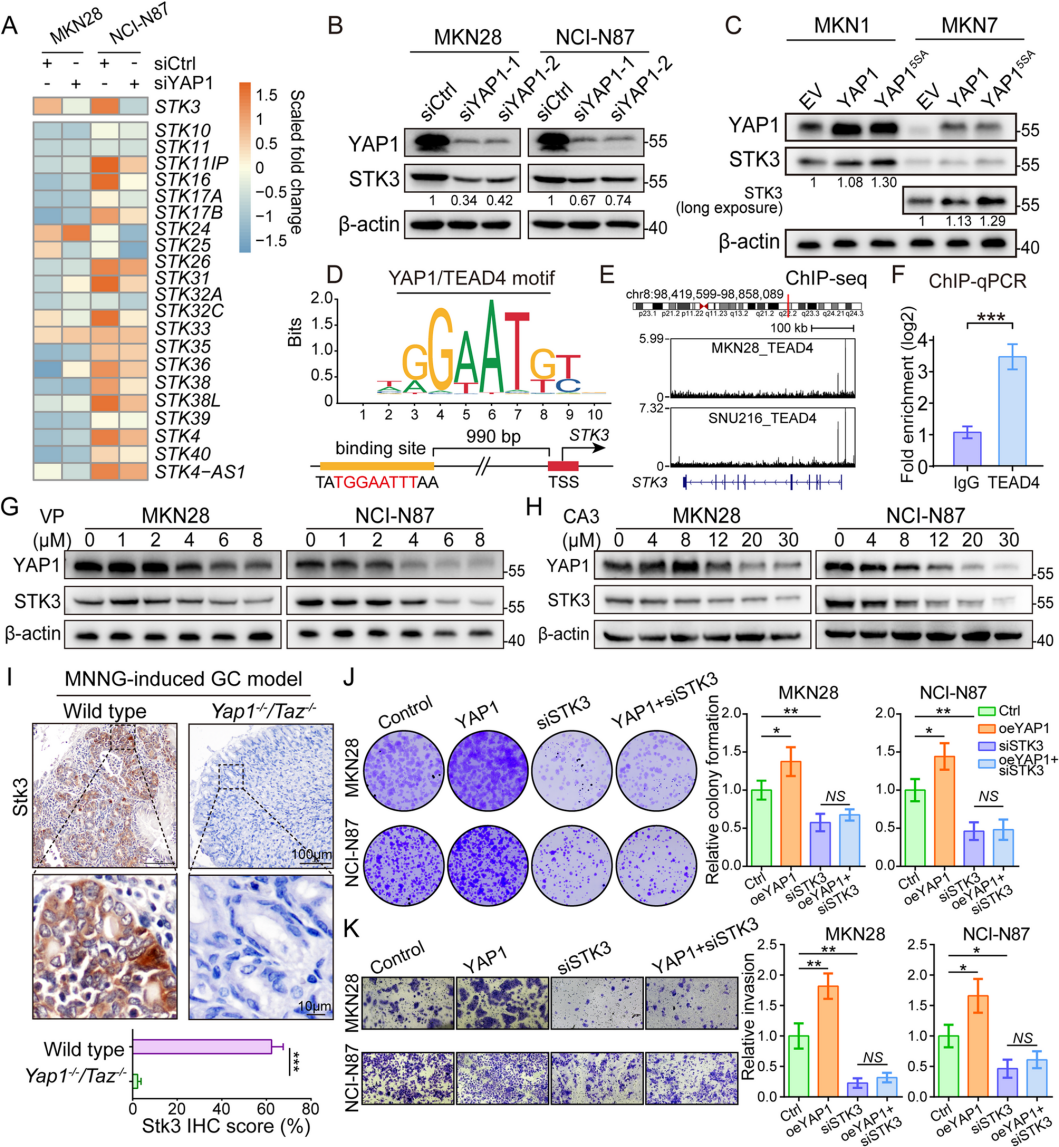
STK3 is transcriptionally regulated by YAP1-TEAD4. (**A**) RNA-seq analysis based on YAP1-deleted cell lines. (**B**) Western bolt revealed the downregulation of STK3 protein expression level after YAP1 knockdown in MKN28 and NCI-N87. (**C**) STK3 level was upregulated after YAP1 overexpression in MKN1 and MKN7 cells. (**D**) A potential YAP1/TEAD4 binding motif was identified in *STK3* promoter region. (**E**, **F**) YAP1-TEAD4 could directly bind to the promoter region of *STK3* and exaggerate its expression, which is confirmed by public ChIP-seq dataset analysis and ChIP-qPCR assay. (**G**, **H**) Both VP and CA3 could downregulate the protein expression level of STK3. (**I**) Stk3 expression was significantly downregulated in *Yap1*^*−/−*^*Taz*^*−/−*^ transgenic mice. (**J**, **K**) STK3 knockdown impaired the colony formation and invasion capacity of MKN28 and NCI-N87, while overexpressing YAP1 failed to rescue the detriment. (*, *P* < 0.05; **, *P* < 0.01; ***, *P* < 0.001)

RNA-seq analysis was conducted on YAP1-deleted GC cell lines to identify novel YAP1 targets. And the results highlighted *STK3* as the sole downregulated STK in both MKN28 and NCI-N87 (Fig. 1A). The knockdown of YAP1 downregulated STK3 expression in GC cell lines with high basal level of YAP1 at both protein and mRNA level (Fig. 1B, Supplementary Fig. S1), whereas the overexpression of YAP1 led to an increase of STK3 expression in YAP1 lowly expressed cell lines MKN1 and MKN7 (Fig. 1C). According to JASPER and EPD, a potential YAP1/TEAD4 binding motif was identified in the promoter region of the *STK3* gene, 992 bps upstream of the transcription start site (Fig. 1D). A peak representing the TEAD4 binding site was identified in the promoter region of *STK3* gene in GC cell lines MKN28 and SNU216 (Fig. 1E). Further validation of the ChIP-seq prediction was then conducted by ChIP-qPCR assay. (Fig. 1F). Two well-known YAP1 inhibitors, verteporfin (VP) and CA3 (CIL56), were demonstrated to effectively reduce the expression levels of both YAP1 and STK3 in the GC cell lines MKN28 and NCI-N87 (Fig. 1G-H). Regarding mice models, *Yap1*^*−/−*^*Taz*^*−/−*^ transgenic mice were treated with Methylnitronitrosoguanidine (MNNG) oral delivery for 6 months to generate GC. And the expression level of Stk3 was significantly reduced in *Yap1/Taz* deficient mice (Fig. 1I). Rescue assays were subsequently conducted to ensure the engagement of STK3 in the YAP1-mediated oncogenic function. As revealed by the invasion and colony formation assays, overexpression of YAP1 led to exaggerated oncogenic phenotypes of GC cell line MKN28 and NCI-N87, while STK3 depletion inhibited the malignant features. However, the upregulation of YAP1 was unable to activate cell proliferation and metastasis when STK3 was maintained at a low level (Fig. 1J-K, Supplementary Fig. S2). Further exploration of the potential feedback effect of STK3 on canonical Hippo signaling pathway was conducted in STK3-deleted GC cells. Western blot analysis revealed a decrease in nuclear YAP1 expression, while phosphorylated YAP1, which is primarily present in the cytoplasm, was upregulated (Supplementary Fig. S3). These results indicate a deactivation of YAP1 signaling, which was further proved by the reduced expression levels of YAP1 and CTGF (Supplementary Fig. S4).

**Fig. 2:**
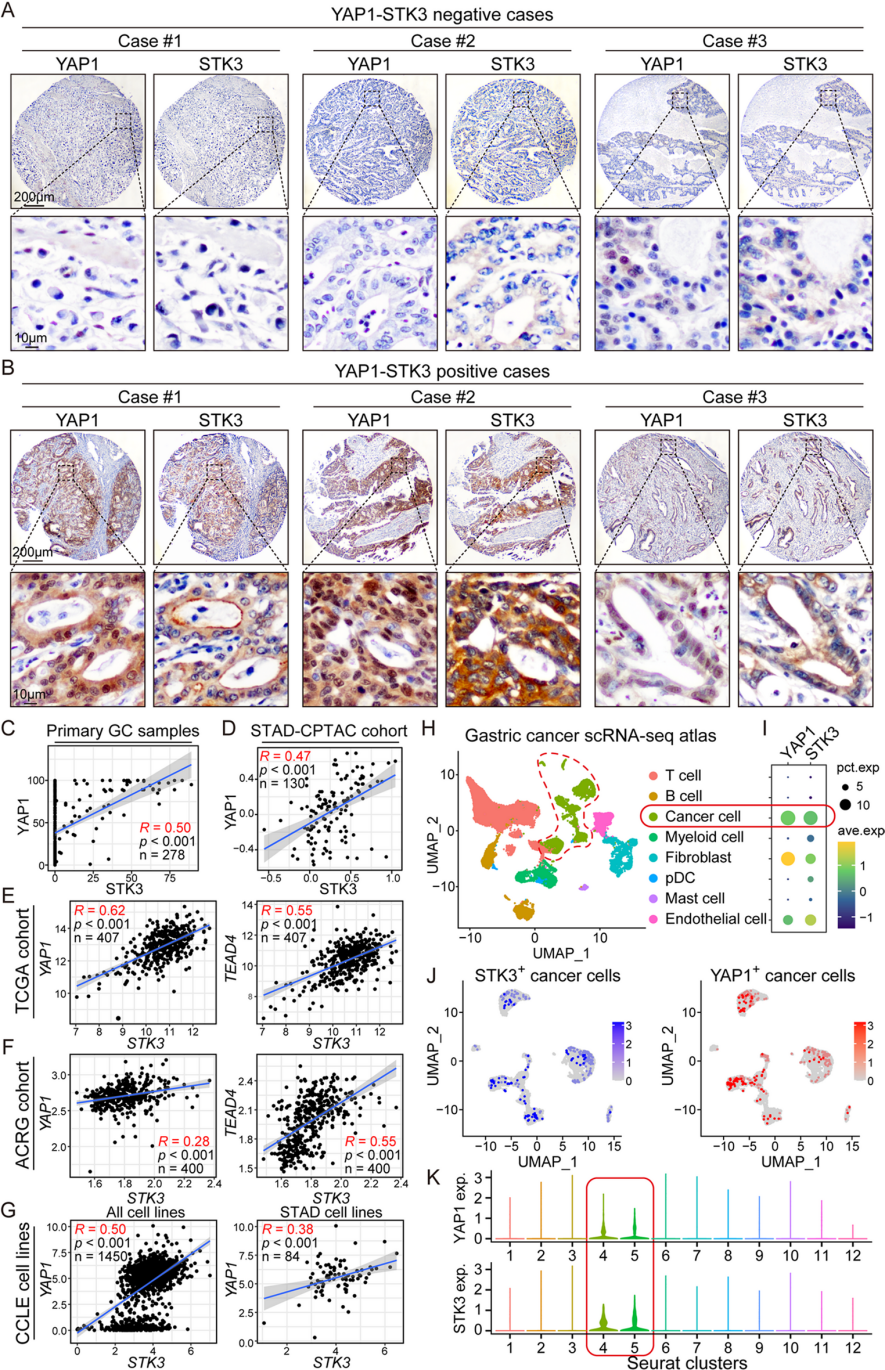
STK3 shows co-upregulation with YAP1 in tumor tissues and cell lines across various GC cohorts. (**A**, **B**) Representative IHC images of YAP1-STK3 dual-negative and dual-positive GC primary samples. (**C**, **D**) Co-upregulation of YAP1 and STK3 was observed in Hong Kong cohort and proteome dataset STAD-CPYAC with a significant correlation. (**E**, **F**) The mRNA level of *STK3* was significantly correlated with both *YAP1* and *TEAD4* expression in TCGA and ACRG cohort. (**G**) The mRNA expression of *YAP1* and *STK3* was remarkably associated in both all cancer cell lines and STAD cell lines. (**H**) UMAP reduction and cell type classification of a public GC scRNA-seq atlas. (**I**) The scRNA-seq data revealed that *YAP1* and *STK3* were largely overexpressed in cancer cells. (J-K) *STK3*^+^ and *YAP1*^+^ cancer cells were clustered in the same sub-clusters

Serial sections of GC primary samples were stained by YAP1 and STK3, and the results demonstrated the co-expression of them in the same regions. As for the subcellular localization, YAP1 upregulation was accumulated in nuclei, while STK3 expression was predominantly localized in the cytoplasm (Fig. 2A-B). Statistically, the protein expression levels of YAP1 and STK3 were correlated in our in-house primary GC cohort (*R* = 0.50) and public proteomic databases CPTAC (*R* = 0.47) (Fig. 2C-D). Two commonly used databases, namely TCGA and ACRG, were adopted to investigate the correlation between *STK3* and *YAP1/TEAD4* expression at mRNA levels. The findings further substantiated the positive correlation between the expression levels of *STK3* and *YAP1*, indicated by Pearson’s R values of 0.62 and 0.28, respectively. Additionally, the expression of *STK3* and *TEAD4* exhibited a positive correlation in two TCGA and ACRG cohorts, with Pearson’s R values of 0.55 and 0.55, respectively (Fig. 2E-F). A close but not significant correlation with poor survival rate was identified in the *STK3-YAP1* co-overexpression group in TCGA cohort (*P* = 0.094) (Supplementary Fig. S5). Meanwhile, Co-upregulation of *STK3* and *YAP1* was also identified in both all cancer cell lines (*R* = 0.50) and STAD cell lines only (*R* = 0.38) (Fig. 2G). Public GC scRNA-seq dataset was adopted, and the results illustrated that *STK3* and *YAP1* upregulation were largely enriched in the cancer cells within GC TME (Fig. 2H-I). By further subgrouping the cancer cells, the *STK3*^*+*^ and *YAP1*^*+*^ cells were classified into the same Seurat clusters (cluster 4 and 5) (Fig. 2J-K).

**Fig. 3:**
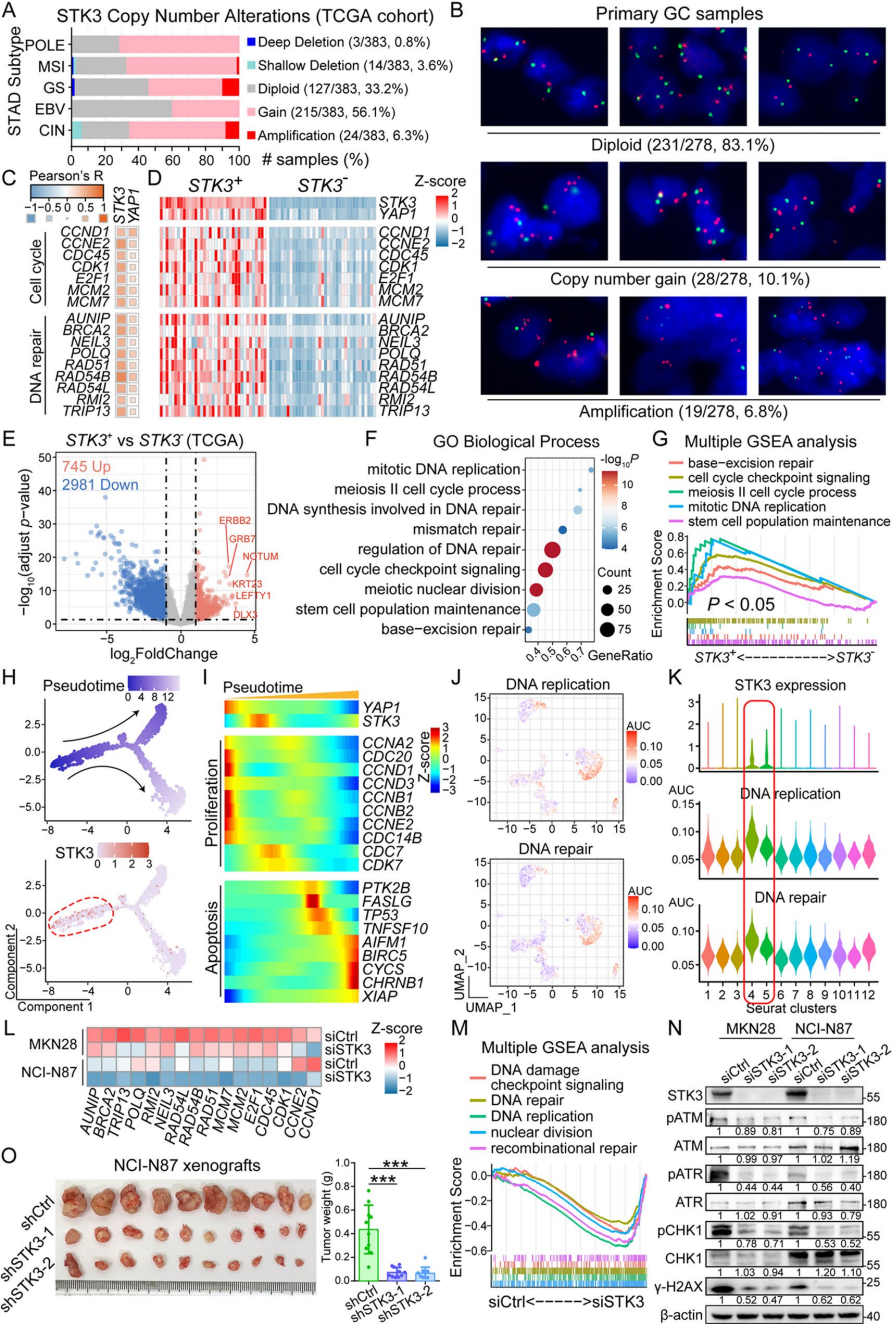
*STK3* gene is amplified in GC tissues, playing a crucial role in the regulation of cell cycle and DNA damage repair progression. (**A**) Copy number gain of STK3 gene was widely observed crossing all molecular subtypes of TCGA-STAD cohort (POLE, polymerase epsilon-mutated and ultramutated; MSI, microsatellite instability-high; GS, genomically stable; EBV Epstein-Barr virus-positive; CIN, chromosomal instability). (**B**) FISH assay demonstrated the frequent occurrence of *STK3* gene copy number gain and amplification in primary GC samples. (**C**) The mRNA level of *STK3* and *YAP1* were remarkably correlated with the expression of cell cycle markers and DNA repair regulators in TCGA-STAD cohort. (**D**) The expression of cell cycling and DNA repair signatures are significantly upregulated in *STK3*^*+*^ samples when comparing with STK3^−^ samples. (**E**) A volcano plot illustrating different expressed genes between *STK3*^*+*^ and STK3^−^ samples. (**F**, **G**) Enrichment analysis and GSEA highlighted the hyperactivation of cell cycling- and DNA damage repair-related biological processes in *STK3*^*+*^ samples. (H, I) Pseudotime analysis on the scRNA-seq data indicated that STK3 was upregulated in the early stage of cancer cell evaluation, along with cell proliferation gene markers. (**J**, **K**) GSVA indicated a higher activity of DNA replication and DNA repair pathways in *STK3*^*+*^ cells. (**L**, **M**) RNA-seq analysis on STK3-deleted GC cell lines demonstrated declined expression of cell cycling/DNA repair-related gene expression and impaired pathway activities. (**N**) Phosphorylation of ATM, ATR, CHK1 and the expression of γ-H2AX were downregulated by STK3 depletion. (**O**) STK3 depletion impaired the xenograft formation capacity of NCI-N87 cells (*n* = 10 mice/group; ***, *P* < 0.001)

A large proportion of GC samples (56.1%) in the TCGA cohort showed *STK3* copy number gain among all five molecular subtypes. Several cases in three specific subtypes, namely microsatellite instability-high (MSI), genomically stable (GS), and chromosomal instability (CIN), demonstrated *STK3* amplification. Besides, only 3 cases of GS subtype showed *STK3* deep deletion (Fig. 3A). In our in-house GC cohort, 10.1% cases demonstrated *STK3* copy number gain, and 6.8% of the samples showed *STK3* amplification (Fig. 3B). The expression level of multiple genes involved in cell cycle and DNA repair progress were closely correlated with the mRNA level of both *STK3* and *YAP1* (Fig. 3C). Meanwhile, the same gene set showed opposite expression patterns between *STK3*^*+*^ and *STK3*^*−*^ samples (Fig. 3D). Volcano plot demonstrated that multiple cell cycling- and DNA repair-related genes were significantly upregulated in the *STK3*^*+*^ samples, while several notorious oncogenes were highlighted with significant and relatively higher upregulation in *STK3*^*+*^ samples, such as *ERBB2*,* GRB7*,* NOTUM*,* KRT23*,* LEFTY1*, and *DLX3* (Fig. 3E). Further enrichment analysis revealed several cell cycling and DNA repair processes, such as mismatch repair, base-excision repair, and cell cycle checkpoint signaling, were activated in the *STK3*^*+*^ samples (Fig. 3F-G). Pseudotime analysis on the scRNA-seq data indicated that *STK3* expression was chronologically correlated with cell proliferation regulators, whereas the activation of apoptosis mediators was enriched in the other end of pseudotime schedule (Fig. 3H-I). GSVA highlighted two pathways, namely DNA replication and DNA repair, were both hyper-activated in *STK3*^*+*^ cells (Fig. 3J). Besides, the average AUC score of these two pathways were relatively higher in the *STK3*^*+*^ Seurat clusters (Fig. 3K). To validate the oncogenic function of STK3 inferred from public databases, RNA-seq was conducted, revealing downregulated expression of cell cycle regulators and the decrease of cell cycle- and DNA repair-related pathway activities (Fig. 3L-M). Western blot assay revealed a downregulated phosphorylation level of ATM, ATR, and CHK1 after STK3 depletion, indicating a reduced activity of DNA repair in GC cell lines (Fig. 3N). In xenograft models, mice with subcutaneous injection of shSTK3-treated NCI-N87 cells generated significantly smaller tumors (Fig. 3O).

**Fig. 4:**
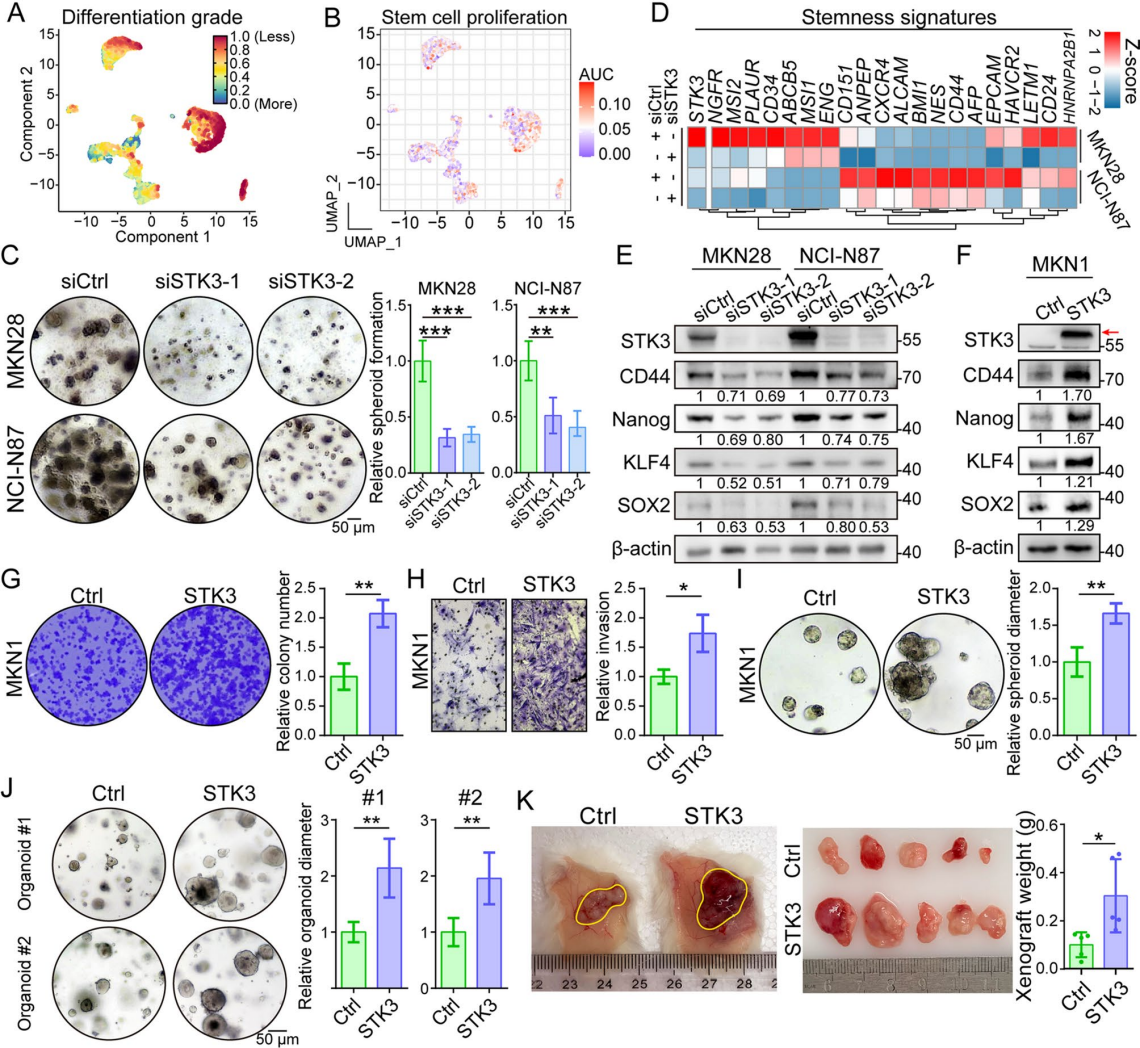
STK3 facilitates the acquisition of stemness features in GC cells, consequently accelerating tumor formation. (**A**, **B**) *STK3*^+^ cancer cells demonstrated less differentiation grade and higher activity in stem cell proliferation. (**C**) Knockdown of STK3 impaired the spheroid formation ability of MKN28 and NCI-N87. (**D**) STK3-depletion downregulated the mRNA levels of multiple stemness signatures. (**E**) Western blot assay indicated downregulated protein expressions of stemness markers in STK3-deleted cells, such as CD44, Nanog, KLF4, and SOX2. (**F**) Overexpression of STK3 increased the expression of CD44, Nanog, KLF4, and SOX2. (**G**-**I**) STK3 overexpression significantly enhanced the colony formation, invasion, and spheroid formation ability of MKN1 cells. (**J**, **K**) STK3 overexpression accelerated the growth of patient-derived organoids and cell line-derived xenografts. (*n* = 5 mice/group; *, *P* < 0.05; **, *P* < 0.01; ***, *P* < 0.001)

Besides the regulatory function of STK3 on cell cycling and DNA-repair, former analysis also indicated the potential of STK3 in maintaining the population of stem cell (Fig. 3G). CytoTRACE analysis revealed that *STK3*^*+*^ cells exhibited a lower differentiation grade, suggesting higher stemness features (Fig. 4A), which is consistent with GSVA scoring on the stem cell proliferation pathway (Fig. 4B). Experimental results indicated that knocking down of STK3 significantly inhibited the spheroid formation capacity of MKN28 and NCI-N87 (Fig. 4C). Multiple stemness signatures, such as *CD44* and *LETM1*, were co-downregulated in STK3-deleted GC cell lines (Fig. 4D). At protein level, stemness markers including CD44, Nanog, KLF4, and SOX2 were both downregulated by STK3 depletion (Fig. 4E). And the expression of the same stemness markers increased following the overexpression of STK3 in MKN1, a GC cell line which was proved to possess a low baseline level of STK3 (Fig. 4F). Furthermore, malignant features of MKN1, such as colony formation, invasion, and spheroid formation, were all over-activated by STK3 overexpression (Fig. 4G-I). The *in vivo* validation of the oncogenic function of STK3 was subsequently conducted using organoid and mouse models, which resulted in accelerated growth of organoids and subcutaneous xenografts in the STK3-overexpressing groups. (Fig. 4J-K).

**Fig. 5:**
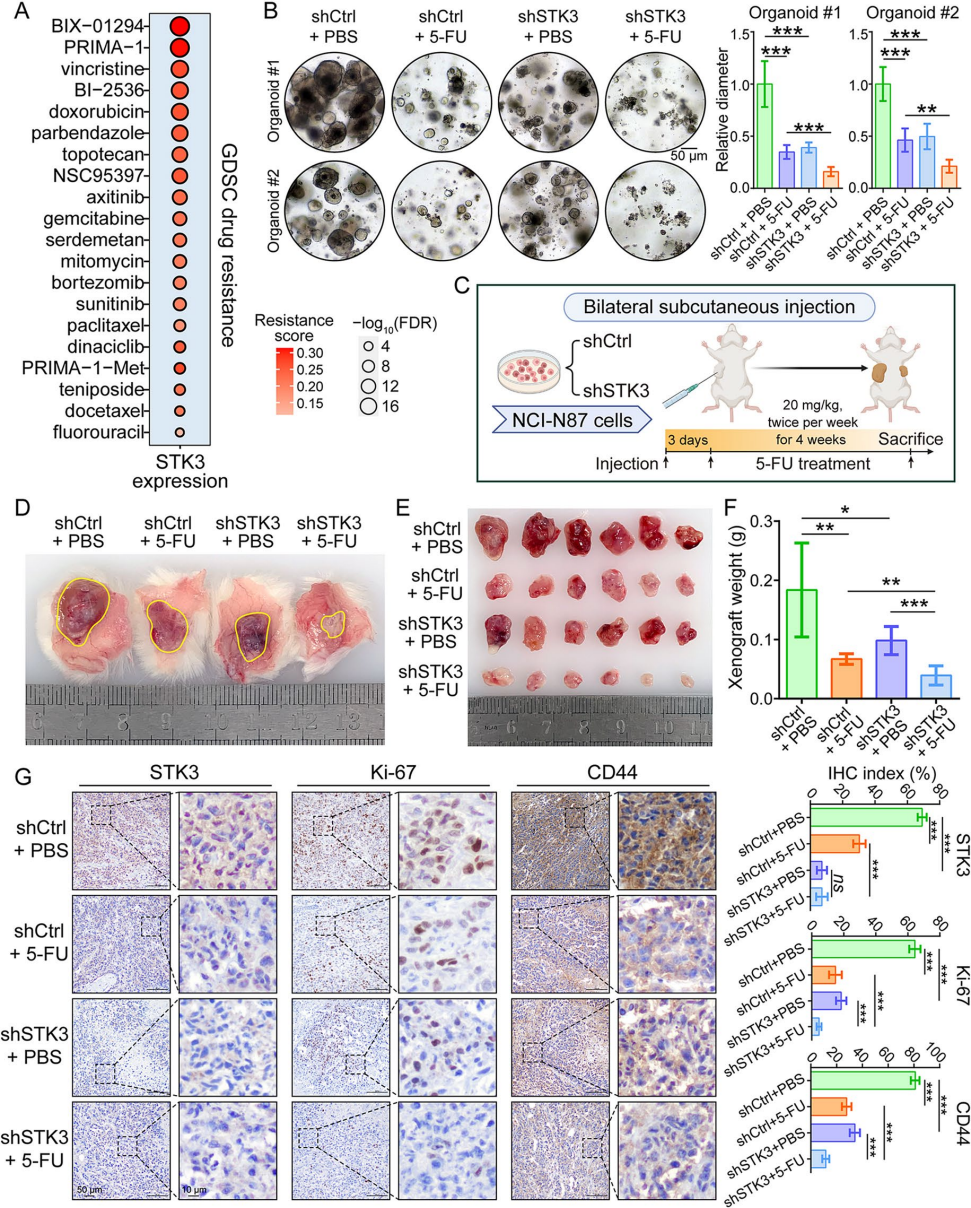
Knockdown of STK3 remarkably improves the GC chemotherapy sensitivity. (**A**) High STK3 expression level is correlated to the resistance of various chemotherapy drugs in GDSC dataset. (**B**) STK3 knockdown enhanced the sensitivity of GC organoids to 5-FU treatment, as indicated by the smaller organoid sizes in shSTK3 + 5-FU group. (**C**) Schematics of the *in vivo* drug sensitivity assay. (**D**) Representative photos of subcutaneous xenografts. (**E**, **F**) Xenograft formation was significantly restrained in STK3-deletion group and demonstrated heightened sensitivity to 5-FU treatment (*n* = 6 mice/group). (**G**) Representative IHC images of xenograft sections stained by STK3, cancer cell proliferation marker Ki-67, and stemness marker CD44 (*ns*, not significant; *, *P* < 0.05; **, *P* < 0.01; ***, *P* < 0.001)

The STK3-mediated biological processes, such as DNA repair and cancer cell stemness maintenance, are closely correlated with chemotherapy resistance [29, 30]. In the GDSC drug resistance database, STK3 expression was highly correlated with the resistance against multiple chemotherapy drugs (Fig. 5A). Two organoids were adopted for investigating the role of STK3 in regulating drug sensitivity. As the results suggested, the organoid growth was inhibited by 5-FU treatment or STK3-depletion alone, and the co-administration group demonstrated significant decrease of organoid sizes when compared with 5-FU treatment group (Fig. 5B). In the mice models, NSG mice were subcutaneously injected with NCI-N87 cells treated by shCtrl of shSTK3. After a 4-week treatment of 5-FU or PBS, cell-derived xenografts were harvested, weighted, and possessed for further IHC staining (Fig. 5C). Consistently with the organoid assay results, knocking down of STK3 significantly increased the anti-tumor efficiency of 5-FU, which was represented by the largely limited xenograft formation in mice of co-administration group (Fig. 5D-F). As indicated by the IHC staining results, the expression level of cell proliferation marker Ki-67 and stemness marker CD44 were downregulated in 5-FU treatment and shSTK3 groups. More importantly, the co-treatment of 5-FU and STK3-deletion showed superior effectiveness on inhibiting cancer cell proliferation and stemness (Fig. 5G).

**Fig. 6:**
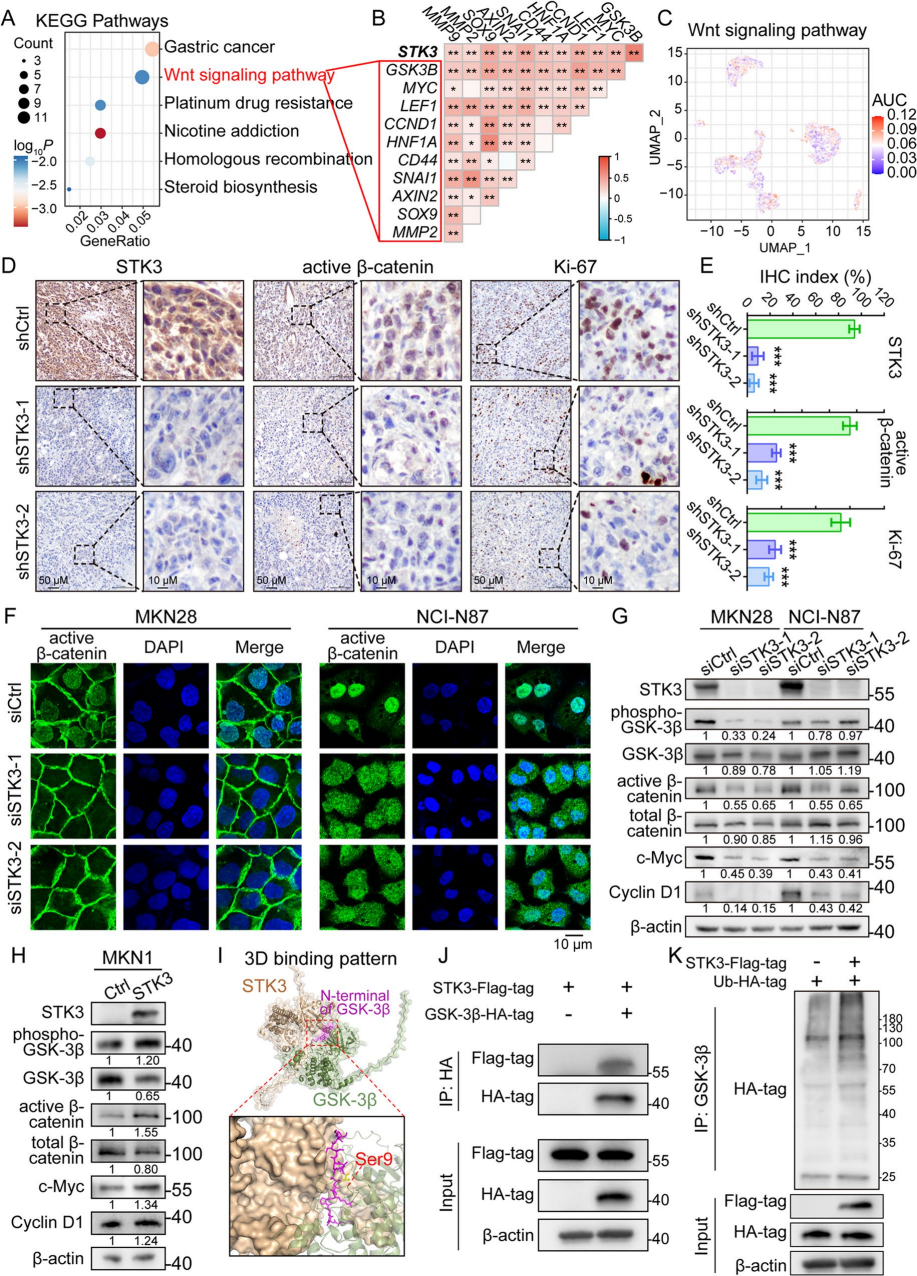
STK3 mediates Wnt signaling transduction by directly interacting with GSK-3**β.** (**A**) Wnt signaling pathway was highlighted by enrichment analysis. (**B**) STK3 mRNA expression was positively correlated with multiple Wnt signaling signatures. (**C**) STK3 positive cells presented higher Wnt signaling activity. (**D**, **E**) Representative IHC images of xenograft sections stained by STK3, active β-catenin, and Ki-67. (**F**) Knockdown of STK3 inhibited the nuclear accumulation of active β-catenin. (**G**) STK3 depletion inhibited the phosphorylation of GSK-3β, leading to the upregulation of β-catenin degradation and downregulation of Wnt signaling signatures. (**H**) Overexpression of STK3 upregulated the phosphorylation of GSK-3β, and the expression of Wnt signaling markers. (**I**) The predicted binding pattern of STK3 and GSK-3β. (**J**) Co-IP assay proving the direct interaction between STK3 and GSK-3β. (**K**) introduction of STK3 accelerated the ubiquitination process of GSK-3β (***, *P* < 0.001)

The KEGG pathway enrichment analysis on the TCGA cohort revealed that Wnt signaling pathway was the most upregulated pathway in *STK3*^*+*^ samples (Fig. 6A). Besides, the expression of *STK3* and multiple Wnt signaling regulators and effectors, such as *GSK3B*, *MYC*, *CCND1*, and *SNAI1*, were significantly correlated (Fig. 6B). To add up with, the *STK3*^+^ cancer cells in scRNA-seq atlas also demonstrated relatively higher activity of Wnt signaling (Fig. 6C, Supplementary Fig. S6). The activity of Wnt signaling and cell proliferation were investigated by IHC staining, which indicated that the inhibition of STK3 expression largely decreased the expression of active β-catenin and Ki-67 (Fig. 6D-E). Immunofluorescence was then conducted on two GC cell lines to demonstrate the impact of STK3 on Wnt signaling activity. As revealed by the results, the active β-catenin was largely accumulated in the nucleus of cancer cells in regular conditions, executing its oncogenic function. After STK3 deletion, the nuclear active β-catenin was dismissed and then distributed to the cytoplasm, indicating the dysfunction of Wnt signaling (Fig. 6F). Furthermore, overexpression of β-catenin partially rescued the inhibitory effect of STK3 depletion on the cancer cell stemness acquisition and GC organoid growth (Supplementary Fig. S7). Western blot results revealed that the phosphorylated GSK-3β, an upstream regulator of the phosphorylation of β-catenin, was significantly downregulated after knocking down of STK3, along with the active β-catenin, c-Myc, and Cyclin D1. Meanwhile, the expression level of GSK-3β and β-catenin remain undisturbed (Fig. 6G). In MKN1 cells, overexpressing STK3 would remarkably activate the phosphorylation of GSK-3β, and subsequently the expression of Wnt signaling markers c-Myc and Cyclin D1 (Fig. 6H). To demonstrate the direct interaction between STK3 and GSK-3β, AlphaFold 3 was adopted and the predicted binding pattern showed close affinity between STK3 and the N-terminal of GSK-3β, which includes the phosphorylation site that inactivates GSK-3β (Ser 9) (Fig. 6I). Further co-immunoprecipitation (Co-IP) assay demonstrated that STK3 could directly bind to GSK-3β and induce the ubiquitination of it (Fig. 6J-K). The *in vitro* kinase assay results demonstrated that the proposed binding and phosphorylation-dependent regulation could be reconstituted in a purified system without cellular components. This was evidenced by the increased ATP consumption in the STK3-GSK-3β co-existence system compared with the STK3-only or GSK-3β-only systems (Supplementary Fig. S8). Functional assays demonstrated that STK3 overexpression exhibited no regulatory effect on the malignance of MKN1 cells when co-transfected with S9A mutated GSK-3β. However, overexpression of STK3 partially rescued the proliferation and invasion ability in cells with overexpression of unmutated GSK-3β. (Supplementary Fig. S9).

**Fig. 7:**
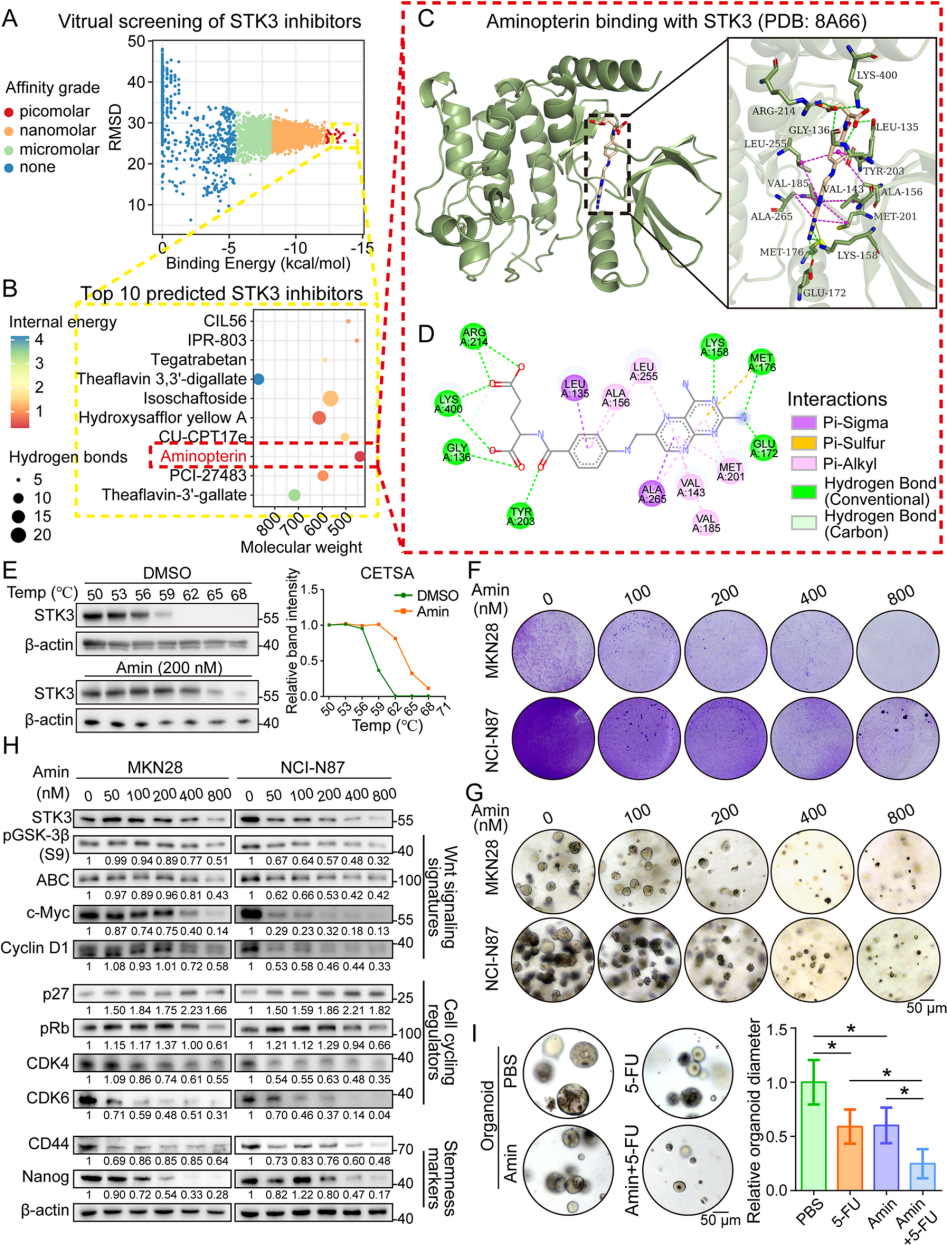
Aminopterin is screened out by in silico modeling as a highly promising inhibitor for STK3. (**A**-**B**) Amin was identified as the most effective STK3 inhibitor among 4511 candidates with anti-tumor properties. (**C**-**D**) Predicted binding pattern and intermolecular interactions of Amin-STK3 complex (**E**) CETSA was conducted to further prove the binding between Amin and STK3. (**F**, **G**) Colony and spheroid formation ability of GC cell lines were restrained by Amin treatment in a dose-dependent manner. (**H**) Amin treatment downregulated the expression level of Wnt signaling signatures, cell cycling regulators, and stemness markers. (**I**) Amin administration impaired the growth of GC organoids and enhanced their sensitivity to 5-FU treatment (*, *P* < 0.05)

High throughput virtual screening was performed on an anti-tumor chemical library to identify novel inhibitors of STK3. Ranked by binding affinity and referring to the root mean square deviation (RMSD) of binding conformation, the top 10 candidates were screened out for further evaluation (Fig. 7A-B). Finally, aminopterin was selected out for its advantages in molecular weight, internal stability, and the composition of intermolecular interactions (Fig. 7C-D). The cellular thermal shift assay (CETSA) revealed that STK3 protein would be largely degraded when the sample was heated to 59 ℃, while aminopterin treatment remarkably increased the stability of STK3 protein, indicating the direct binding between STK3 and aminopterin (Fig. 7E). The *in vitro* kinase assay results demonstrated a significant inhibitory function of aminopterin on the STK3 kinase activity (Supplementary Fig. S10) Functional assays proved that aminopterin treatment could inhibit the malignant features of GC cell lines, such as colony and spheroid formation, in a dose-dependent manner (Fig. 7F-G). As indicated by the dose-dependent expression variation of corresponding markers, aminopterin treatment succeeded in inhibiting STK3 expression and the oncogenic functions activated by it, including Wnt signaling transduction, cell cycling, and stemness maintenance (Fig. 7H). Furthermore, the co-administration of aminopterin and 5-FU regained a better anti-tumor effect than 5-FU monotherapy in our organoid models (Fig. 7I) and mice models (Supplementary Fig. S11). The IHC staining results demonstrated significate downregulation of active-β-catenin and Ki-67 while the expression level of cleaved-PARP was upregulated following both aminopterin treatment alone and in combination with 5-FU (Supplementary Fig. S12). As for the safety profile, administration of aminopterin demonstrated limited impact on mice body weight (Supplementary Fig. S13). Hematoxylin and eosin (H&E) staining results also demonstrated no severe histopathological changes in the major organs of control and treatment groups (Supplementary Fig. S14).

## Discussion

Until now, GC remains a major threat to human health. Especially in Asia, due to the consistent concerns in the high salt/oil diet and high-pressure living patterns, the occurrence of GC is shifting to younger generation [31]. Among all kinds of biological pathways involved in tumorigenesis, the Hippo pathway is notorious for its predominantly regulatory function in cancer cell malignancy and cross-talks with other oncogenic pathways. Studies have demonstrated that the high expression level and nuclear transition of YAP1 would induce the enhancement of multiple aggressiveness features of cancer cells, such as proliferation, migration and cancer stem cell population maintenance [32, 33]. Some studies also highlighted that Hippo pathway activity can directly mediate the ferroptosis status within cancer cells [34]. Mechanistically, the regulation of Hippo pathway on carcinogenesis is mainly facilitated by the regulation on YAP1. In the Hippo-off status, the phosphorylation cascade Mst1/2-Lats1/2 would be deactivated, and unphosphorylated YAP1 was transported into the nucleus, binding to TEADs directly to facilitate its oncogenic roles. Subsequently, the co-transcription factor would activate the expression of multiple oncogenic genes, such as CTGF and CYR61, to encourage tumorigenesis [35, 36]. The YAP1-5SA mutation, in which five serine residues (S61, S109, S127, S164, and S397) are substituted with alanine, represents a constitutively active form of YAP1 that functionally mimics YAP1 overexpression [37, 38]. These five serine residues serve as the primary phosphorylation sites for Lats1/2 kinases within the Hippo pathway. By abolishing these critical phosphorylation sites, the 5SA mutant is capable of escaping Hippo cascade-mediated inhibitory phosphorylation. Functionally, this mutant recapitulates the phenotypic effects of YAP1 overexpression, including promotion of cell proliferation, epithelial-mesenchymal transition, and other oncogenic processes, while remaining unresponsive to normal Hippo pathway regulation.

In this work, we identified STK3 as a novel and indispensable YAP1 downstream target which could largely utilize the oncogenic role of YAP1. Starting from a coincident observation, we found that YAP1-deleted GC cells and CAFs demonstrated concordant and significant decrease in *STK3* expression. We then further demonstrated the co-downregulation in protein level by siRNA-mediated YAP1 knockdown, YAP1 inhibitor treatment, and *Yap1*^*−/−*^*Taz*^*−/−*^ transgenic mice models, and all the results proved that the downregulation of YAP1 can inhibit STK3 expression. Until now, the hypothesis generated from the data is a totally reverse against former knowledge, as STK3, also named as Mst2, is long-believed and well-proven to be an upstream inhibitor of YAP1. To further validate the realness of the regulation between YAP1 and STK3, we conducted correlation analysis on multiple databases. The results strongly supported that the expression of YAP1 and STK3 are significantly and positively correlated, no matter in primary samples or cancer cell lines, in mRNA or protein levels, in bulk scale or single cell scale, and in in-house or public databases. Besides, a TEAD4 binding motif was identified in the promoter sequence of *STK3*, and this binding site was further validated by both ChIP-seq and ChIP-qPCR. Taken together, we conclude that STK3 is a direct downstream target gene of YAP1/TEAD4.

Given its promoting function in the YAP1 phosphorylation and degradation process, STK3 is canonically considered as a tumor suppressor gene. However, recent studies have unmasked the oncogenic potential of STK3 in breast and prostate cancer progression [39, 40]. In our previous study, STK3 was also demonstrated to be a promoting factor for the proliferation and migration activity of GC cell lines [41]. Again, we challenged the classic thinking by checking the genome stability of *STK3*, which revealed a notable percentage of *STK3* amplification/copy number gain cases while less than 1% GC patients showed *STK3* deletion. Further analysis of the TCGA-STAD cohort demonstrated a similar pattern of *STK3*, which indicates a strong oncogenic potential of this traditionally recognized tumor suppressor gene. As we dig deeper into the expression pattern of STK3, it was observed that while *STK3* amplification was noted in a limited group of patients, the overexpression of STK3 is more commonly observed in GC patients: In the TCGA cohort, upregulated *STK3* levels were identified in 76 GC patients (19.8%), whereas only 24 patients (6.3%) exhibited *STK3* gene amplification. Similarly, in our in-house cohort, 19 patients (6.8%) demonstrated *STK3* amplification, while 63 cases (22.7%) showed elevated STK3 levels. These findings suggest that the oncogenic function of STK3 is not solely a result of gene amplification, but is also mediated by upstream mechanisms. This underscores the involvement of the previously established YAP1/TEAD4-STK3 regulatory cascade in the activation of STK3.

The potential negative feedback of STK3 upregulation on YAP1 phosphorylation was also investigated. The canonical tumor suppressor function of STK3 was considered to be largely dependent on regulating the phosphorylation and then degradation of active YAP1. However, we observed a slight downregulation of YAP1 and upregulation of phosphorylated YAP1 in STK3-deleted GC cell lines. Meanwhile, the expression level of CTGF (a classic downstream transcriptional target of YAP1) was also downregulated after STK3-deleption, which reflects a decreased transcription activity of the co-transcription factor YAP1/TEADs. More precisely, the expression level of YAP1 in nuclear was decreased in STK3-deleted cells, while phosphorylated YAP1, which is primarily present in the cytoplasm, was upregulated. All these results suggested that the knockdown of STK3 would not protect active YAP1 from being phosphorylated by Lats1/2 in GC cell lines as canonically hypothesized. In contrast, STK3 deletion led to the decreased activity of the oncogenic YAP1 signaling, which might result from its significant inhibition on the tumor cell malignancy.

Subsequent biological analysis based on the TCGA cohort and scRNA-seq atlas also emphasized the oncogenic potential of STK3 in regulating cell cycling and DNA repair progress. Notably, some stemness-related processes were also included in the enrichment analysis. The maintenance of cancer cell stemness is largely dependent on the activation of a series of stemness regulators. Within this criterion, several stemness markers, namely CD44, Nanog, KLF4, and SOX2, were selected as the predominant ones in GC progress [42, 43]. In the following *in vitro* and *in vivo* validations, STK3 knock-down and overexpression assays harvested significant variations in marker gene expression levels and malignant phenotypes, which coherently meet the former expectations of the oncogenic roles of STK3.

The drug resistance to chemotherapy remains a major challenge in GC treatment, and the detailed molecular mechanisms within this biological mutation are still not thoroughly elucidated due to the complexity and variability of the cancer cell genome [44, 45]. The Hippo-YAP1 signaling has been long identified as an indispensable participator in the occurrence of chemotherapy resistance [46]. As acknowledged, elevated transcriptional activity of YAP1 would significantly enhance the response to DNA repair of cancer cells, which would largely abolish the lethality of chemo drugs. Besides, YAP1-mediated increase of cancer stem cell population also contributed to the recurrence of GC after first-line treatment. Now as STK3 has demonstrated regulatory functions in both DNA repair and cancer stem cell population maintenance, we wondered if the expression level of STK3 can affect the drug sensitivity to chemotherapy. By both patient-derived organoid and cell-derived xenograft models, we observed a better inhibitory effect of 5-FU when co-administrated with shSTK3. As indicated by the IHC staining, the proliferation and cancer stem cell population were impaired by 5-FU treatment, while more severe inhibition was observed in the shSTK3 group. Collectively, we conclude that STK3 overexpression in GC patients could lead to the hyperactivation of malignant phenotypes and resistance to chemotherapy. Mechanistically, the activation of STK3 accelerated the cell cycling and DNA repair response of GC cells while contributing to the maintenance of cancer stem cell population as well.

Although the oncogenic potential of STK3 has been observed in several reports, the deep mechanism of this non-canonical function was hardly explored. By enrichment analysis on the genomic profile of STK3-deleted samples, we found the Wnt signaling pathway ranks as one of the most distributed pathways. As a well-recognized oncogenic factor, this notorious pathway is vital for the genomic activation of multiple oncogenes, especially cancer cell migration and stemness markers. More interestingly, the crosstalk between Hippo and Wnt signaling has been identified in multiple patterns, such as Wnt5A-ROR2-YAP1 [47] and FZD10-Lats1/2-YAP1 axis [48]. Some studies also reported the mutual-indispensable and direct binding of YAP1 and β-catenin in intrahepatic cholangiocarcinoma [49]. Recent works further demonstrated the crosstalk could be established by dual directions, as YAP1 was identified as a crucial promotor for the adrenocortical cancer progression by mediating the transcription activity of β-catenin [50]. Another study focused on the GC hepatic premetastatic niche revealed that the ephrin A1-initialized YAP1-CCL2-Wnt/β-catenin axis bridged the malignant communications between cancer cells and hepatic stellate cells during GC hepatic metastasis [51]. Inspired by the bioinformatic findings, we firstly validated the regulation of STK3 on Wnt signaling activation, and the data proved that the expression of active (non-phosphorylated) β-catenin and nuclear accumulation of β-catenin were consistently decreased after knockdown of STK3. However, the biological function of STK3 is to mediate the phosphorylation of substrate proteins on the serine or threonine residues [52, 53], which indicates the existence of other intermediators within the STK3-β-catenin signaling axis. Among all direct regulators of β-catenin, GSK-3β is particularly manipulated by its phosphorylation status: the phosphorylation at Ser9 of GSK-3β is the most common mechanism of GSK-3β inactivation, which would lead to the deactivation of β-catenin phosphorylation and ubiquitination [54]. To further elucidate the detailed mechanisms within the STK3-Wnt signaling transduction, we conducted both STK3 knockout and overexpression assays in GC cell lines, which demonstrated a hypothesis-endorsing expression variations of Ser9-phosphorylated GSK-3β and non-phosphorylated β-catenin. Furthermore, the Co-IP and ubiquitination assay confirmed the direct binding of STK3 and GSK-3β, which subsequently led to the degradation of GSK-3β. Taken together, these results revealed a novel STK3/GSK-3β/β-catenin signaling axis which was responsible for the oncogenic functions of YAP1-derived STK3 upregulation. Meanwhile, this work also proposed a novel crosstalk between Hippo and Wnt signaling, enriching the understanding of the signaling transduction during GC progression.

Given the high incidence of *STK3* gene amplification in GC patients and its significant effects in promoting oncogenesis and chemo-therapy resistance, there is considerable potential in developing STK3-targeted treatments. Classic Mst1/2 inhibitors like XMU-MP-1 were well developed with good efficiency, appreciable pharmacokinetics, and tolerable side effects, aiming to fulfill the requirements of tissue repair and regenerative medicine [55]. Given the promising records in efficiency and safety profiles of STK3-targeting strategy, coupled with the absence of STK3 inhibitors specially designed for cancer treatment, we employed high throughput virtual screening to identify candidate STK3 inhibitors based on a previously constructed library comprising 4511 chemicals with anti-tumor potentials. After a comprehensive consideration combining the druggability evaluation of small molecules and the stability of binding conformation, aminopterin emerged as the prior candidate and demonstrated significant STK3-inhibiting and tumor-suppressing effects *in vitro*.

Aminopterin, an analogue of folic acid, has long been recognized for its significant anti-tumor effects at relatively low doses. Its application has been widely investigated in clinical trials focusing on patients with endometrial carcinoma, leukemia, and psoriasis [56–58]. Notably, aminopterin exhibits excellent solubility, allowing for substantial dissolution in water. Furthermore, its relatively low molecular weight and acidic structure facilitate both absorption and degradation. However, the toxicity of aminopterin raises major concerns and limitations for clinical use, leading to its discontinuation in routine applications. In mouse models, doses above 0.37 mg/kg resulted in 80% mortality, significantly lower than that observed with another analogue, methotrexate [59]. Given these concerns, we examined the safety profile of aminopterin administration in mice. The results demonstrated that administration of aminopterin significantly inhibited subcutaneous xenograft growth while demonstrating limited impact on mice body weight. Additionally, H&E staining results indicated that both aminopterin alone and in combination with 5-FU exhibited tolerable organ toxicity in tumor-bearing NSG mice, as no severe histopathological changes were observed in the major organs of control and treatment groups, except for slight congestion in kidneys and lungs in the treatment groups. Collectively, these characteristics of aminopterin highlight limitations in its therapeutic window and underscore the need for further investigation into structure-based optimization in the drug discovery process to enhance its therapeutic potential.

## Conclusion

In summary, we demonstrated that STK3, against the common knowledge, is transcriptionally regulated by YAP1, and the high expression of STK3 can promote GC progression by manipulating cancer cell malignance. Meanwhile, STK3 upregulation is vital for cancer stem cell population maintenance and DNA repair progress, and thus contributes to the acquisition of chemo-therapy resistance. Mechanistically, STK3 can directly bind to GSK-3β and facilitate its ubiquitination, which leads to the nuclear accumulation of unphosphorylated β-catenin and then the activation of the oncogenic Wnt signaling signatures. Given the structural feasibility and good safety profiles of targeting STK3, we also delivered a promising candidate for STK3-targeted GC therapy. In conclusion, our findings elucidated the unexplored oncogenic function of STK3 and the underlying mechanism, providing a brand-new insight into the crosstalk between Hippo and Wnt signaling.

## Electronic supplementary material

Below is the link to the electronic supplementary material.

Supplementary Material 1

Supplementary Material 2

Supplementary Material 3

## Data Availability

The datasets used and/or analyzed during the current study are available from the corresponding author on reasonable request.
