# Supplementary material for: STK3 is a transcriptional target of YAP1 and a hub component in the crosstalk between Hippo and Wnt signaling pathways during gastric carcinogenesis
Source: Mol Cancer. 2025 Jul 2;24:186. doi: 10.1186/s12943-025-02391-x (PMC12220525; doi:10.1186/s12943-025-02391-x)
Supplement: Supplementary file 1 — Supplementary Material 1 [file 12943_2025_2391_MOESM1_ESM.pdf]

Supplementary Materials for

STK3 is a transcriptional target of YAP1 and a hub component in the crosstalk between Hippo and Wnt signaling pathways during gastric carcinogenesis

Fuda Xie, Yang Lyu, Bonan Chen, Hoi Wing Leung, Peiyao Yu, Tiejun Feng, Canbin Fang, Alvin H.K. Cheung, Bin Zhou, Jianhui Jiang, Ge Zhang, Dazhi Xu, Liang Li, Chen Jiang, Jianwu Chen, Zhaocai Zhou, Liwei An, Bing Huang, Kangmin Zhuang, Xiaobei Luo, Kam Tong Leung, Ching Hei To, Brigette BY Ma, Chi Chun Wong, William KK Wu, Jun Yu, Ka Fai To, Wei Kang§

§ Corresponding author: Wei Kang (E-mail: weikang@cuhk.edu.hk)

This file includes:

Supplementary Materials and Methods
Supplementary Figure S1-S14

Functional assays

Briefly, cells in the colony formation assay were plated into 6-well plates at a lower density of 1,000 cells per well. After cultured for 10 days, the colonies were stained with 0.4% (w/v) crystal violet (Sigma-Aldrich) and then quantified. Cell invasion assays were performed in Matrigel Invasion Chambers (Corning). GC cells suspended in serum-free medium were added to the inner chamber, and the medium containing 10% FBS was added to the bottom. After incubation for 16-20 hours, invaded cells to the lower membrane surface were stained with 0.4% (w/v) crystal violet and then quantified. The quantification of colony formation and invasion assays were carried out by manual colony/cell counting based on images of three randomly selected views token under 20x objective lens. Spheroid formation assays were constructed at the cell density of 1,000 cells/well. The GC cells were cultured in serum-free Advanced DMEM/F-12 (Life technology), containing B27 (1:50, Life technology), 50 ng/mL EGF (Life technology), 100 ng/mL FGF10 (Life technology) and 1% penicillin/streptomycin (Gibco) in a 24-well plate. Cells were monitored for 14 days to harvest spheroid formation, and the diameters of newly formed spheroids were evaluated.

Real-time quantitative reverse transcription PCR (qRT-PCR)

Total RNA extraction was extracted using RNAiso Plus (Takara). PrimeScript RT Master Mix (Takara) was used for cDNA synthesis. qPCR was performed using TB Green Premix Ex Taq (Takara) according to the manufacturer's instructions. The results were normalized and calculated by using the $2^{-\Delta\Delta Ct}$ method. The sequences of primers for each gene were as follows:

Gene name		Sequence (5' - 3')
STK3	Forward	CTTTGGTCCGATGATTTCACCG
	Reverse	GGATGCTGTAAAAGTTGTGTTGC
ACTB	Forward	ATCATGAAGTGTGACGTGGA
	Reverse	CTCAGGAGGAGCAATGATCT

Western blot analysis

The detailed procedure of sample preparation and Western blot assay were carried out based on general protocol. Nuclear and cytoplasmic protein fractions were isolated using a commercial subcellular protein fractionation kit (Nuclear and Cytoplasmic Protein Extraction Kit, Cat. No. P0028, Beyotime) according to the manufacturer's protocol. Briefly, the cell lysates were extracted by RIPA lysis buffer. The total protein was quantified by BCA Protein Assay Kit (ThermoFisher Scientific), separated by sodium dodecyl sulfate-polyacrylamide gel electrophoresis (SDS-PAGE), and then transferred on to polyvinylidene fluoride (PVDF) membranes. The protein-carrying membranes were blocked for 2 hours in a Tris-buffered saline with Tween (TBST) buffer supplemented with 5% bovine serum albumin (BSA, Millipore) before the overnight incubation at 4℃ with corresponding antibodies. After that, the membranes were washed twice with TBST and incubated for another hour with appropriate horseradish peroxidase (HRP)-conjugated secondary antibodies. Protein bands were visualized using enhanced chemiluminescence.

Chromatin immunoprecipitation (ChIP)

Crosslinked chromatin samples were sheared by a Covaris sonication system (S220) to desired fragments (100-500 bp). The sonicated products were incubated with Magnetic Dynabeads Protein G (10003D, Thermo Fisher) and linked with anti-TEAD4 (AB58310, Abcam) or Normal anti-IgG antibody (#2729, Cell signaling). Based on general protocol, equivalent quantities of IP were set for routine PCR assays using specific primers targeting the region within 100 bp from the predicted binding site. Details of primer for STK3 were listed below: Forward 5'-GTG GTC AGA TTA CAA TGG GAT G-3'; Reverse 5'-TGC CAG GAA GAA TAC TCA AGA A-3'

Co-immunoprecipitation (Co-IP) and ubiquitination assay

After transfection with STK3-Flag and/or GSK-3β-HA plasmid for 24 h, MKN1 cell lysates were collected on ice. Protein A/G magnetic beads (25 μL, HY-K0202,

MedChemExpress) and Anti-HA antibody were added to the lysate, incubated at 4 °C over night under gentle agitation. The beads were washed with washing buffer (50 mM Tris-HCl, 150 mM NaCl, 1% NP-40, 0.5% sodium deoxycholate, 0.1% SDS, pH = 7.4) for five times. Finally, the beads, together with the bonded proteins, were boiled at 95°C for 15 minutes and further analyzed by SDS/PAGE and immunoblot analysis. For the ubiquitination assay, MKN1 cells were transfected with Ub-HA and STK3-Flag plasmids. Cells were pre-treated with MG132 at 10 μM for 2 hours and then lysed on ice. Protein A/G magnetic beads, Anti-GSK-3β antibody and protease and phosphatase inhibitor cocktail (HY-K0013, MedChemExpress) were added into the lysate, and then incubated at 4°C over night. The beads were collected and washed five times. Bound proteins were boiled and analyzed by SDS/PAGE followed by immunoblot analysis.

In vitro kinase assays

The phosphorylating activity of STK3 was quantified by STK3 Kinase Enzyme System (#VA7564, Promega) and ADP-Glo™ Kinase Assay (#V6930, Promega). Briefly, the human recombinant STK3 protein (ab60885, Abcam) was mixed with 1x Reaction Buffer A (200 mM Tris [pH 7.5], 100 mM $MgCl_2$, 0.5 mg/ml BSA, and 50 μM DL-Dithiothreitol (DTT)) and Ultra-Pure ATP in a white 96-well plate. The mixture was then supplemented with the corresponding substrates (myelin basic protein (MBP) or human recombinant GSK-3β protein (ab208324, Abcam)), with or without STK3 inhibitor aminopterin (200 nM), and incubated for 40 minutes. Afterwards, ADP-Glo Reagent was added, followed by another 40-minute incubation. Next, Kinase Detection Reagent was introduced to the mixture, and after a further 40 minutes, the luminescence of each well was recorded using a machine. All incubations in this assay were performed at room temperature.

Cellular thermal shift assay (CETSA)

The harvested cells were freeze-thawed three times using liquid nitrogen for complete

cell lysis. The cell lysate-containing supernatants were centrifuged at 20,000 g for 20 minutes at 4°C, diluted in PBS, and then divided into the Treated group (mixed with 10 μM aminopterin) and the Control group (mixed with an empty vehicle). After a 30-minute incubation at room temperature, the lysates were divided into 50 μL aliquots, and each aliquot was heated to the designated temperatures for 3 minutes using a thermal cycler (Applied Biosystems). The heated lysates were centrifuged at 20,000 g for 20 minutes at 4°C to collect supernatants containing the soluble protein fraction for subsequent Western blot analysis.

Supplementary Figure S1

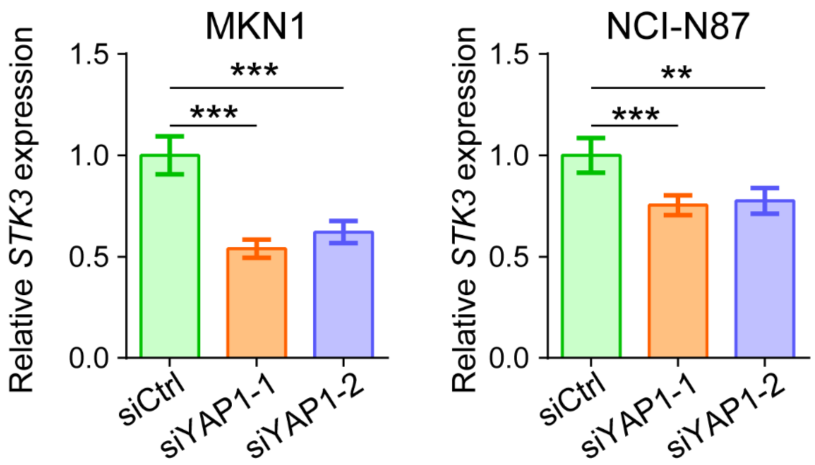

Supplementary Figure S1 The knocking down of YAP1 decreased the mRNA expression level of STK3 in GC cell lines (**, $P < 0.01$; ***, $P < 0.001$).

Supplementary Figure S2

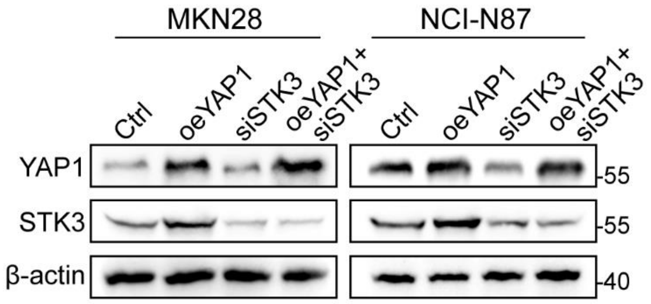

Supplementary Figure S2 The expression level of STK3 and YAP1 after different treatment.

Supplementary Figure S3

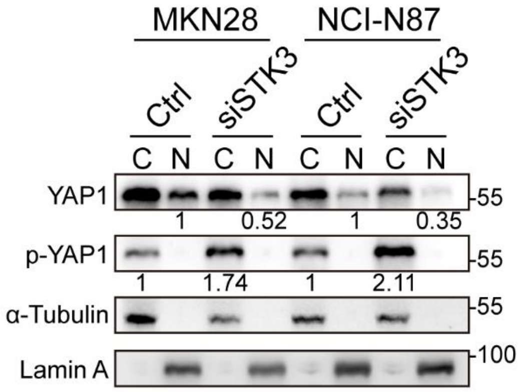

Supplementary Figure S3 STK3-deleted GC cells demonstrated downregulation of YAP1 expression in nuclear and upregulated expression of phosphorylated YAP1 in the cytoplasm.

Supplementary Figure S4

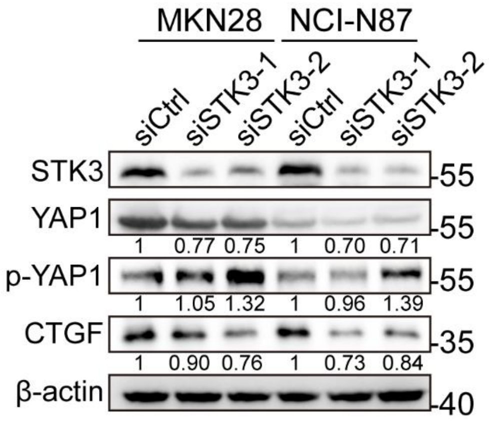

Supplementary Figure S4 STK3 deletion downregulated the activity of canonical YAP1 signaling and the expression levels of the downstream effector CTGF.

Supplementary Figure S5

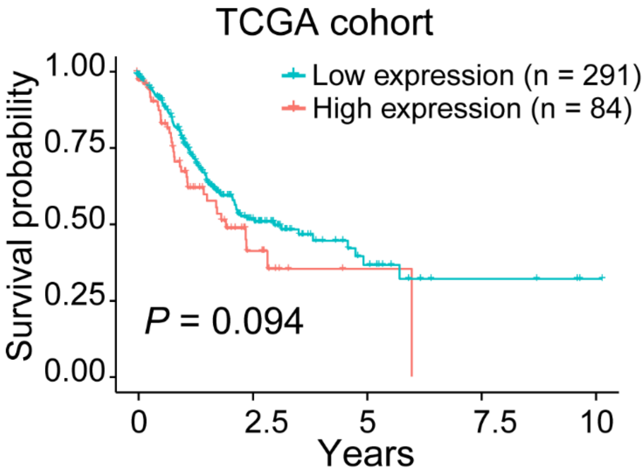

Supplementary Figure S5 A close but not significant correlation with poor survival rate is identified in the high STK3-YAP1co-expression group in TCGA cohort (*P* = 0.094).

Supplementary Figure S6

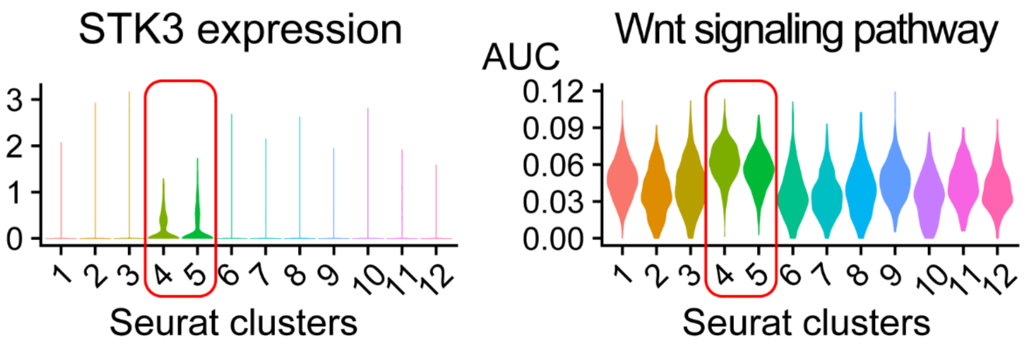

Supplementary Figure S6 GSVA results demonstrated relatively upregulated enrichment scores regarding "Wnt signaling pathway" in the same clusters with high STK3 expression, cluster 4 and 5.

Supplementary Figure S7

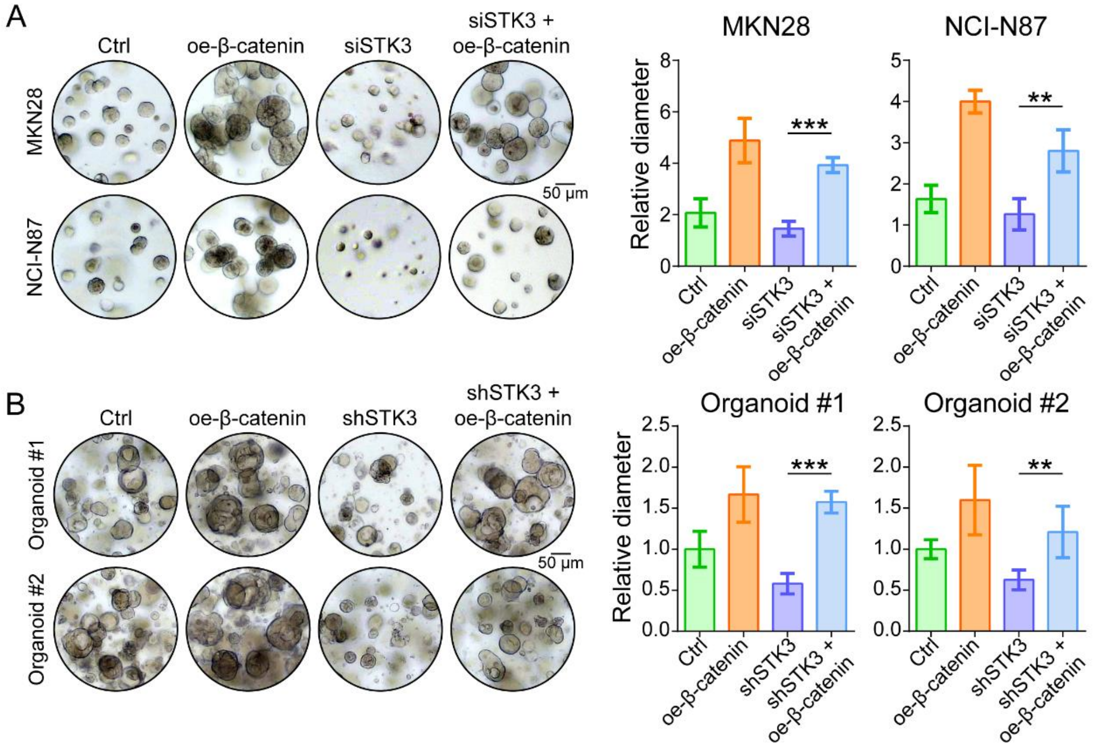

Supplementary Figure S7 β-catenin overexpression partially rescued the inhibitory effect of STK3 depletion on the cancer cell stemness acquisition (A) and GC organoid growth (B) (5 random fields/sample; **, $P < 0.01$; ***, $P < 0.001$).

Supplementary Figure S8

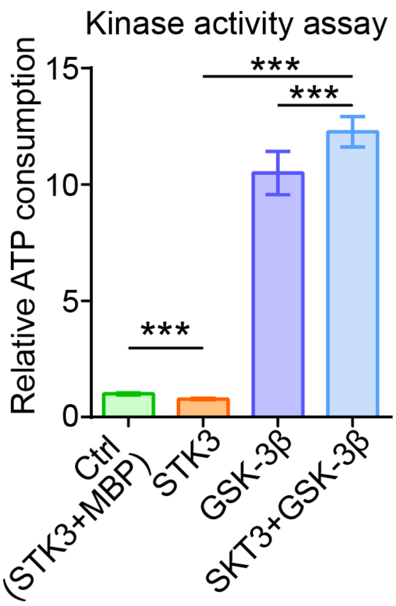

Supplementary Figure S8 ATP consumption was upregulated in the STK3-GSK-3β co-administration system when compared with the STK3-only or GSK-3β-only systems (***, $P < 0.001$).

Supplementary Figure S9

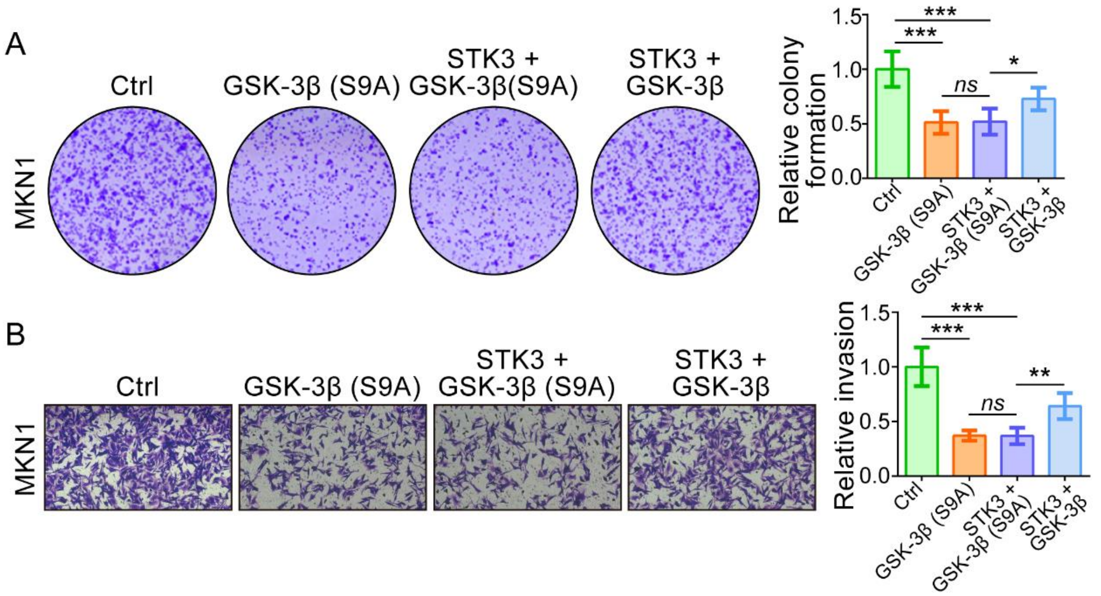

Supplementary Figure S9 Overexpression of STK3 partially rescued the proliferation (A) and invasion ability (B) in cells with overexpression of unmutated GSK-3β, while for the mutated GSK-3β, STK3 overexpression lost the rescue ability on the cell proliferation and invasion. (5 random fields/sample; *ns*, not significant; *, $P < 0.05$; **, $P < 0.01$; ***, $P < 0.001$).

Supplementary Figure S10

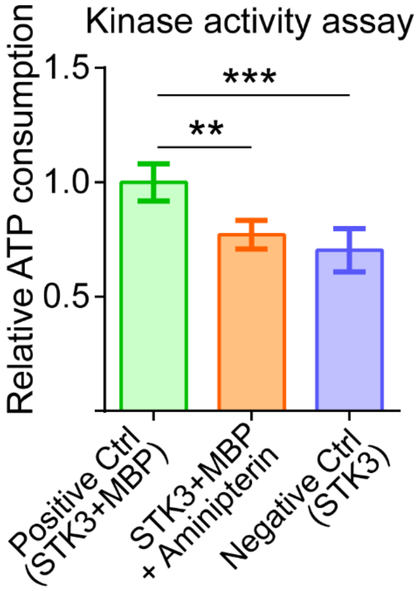

Supplementary Figure S10 Aminopterin administration demonstrated significant inhibition on the phosphorylating activity of STK3 (**, $P < 0.01$; ***, $P < 0.001$).

Supplementary Figure S11

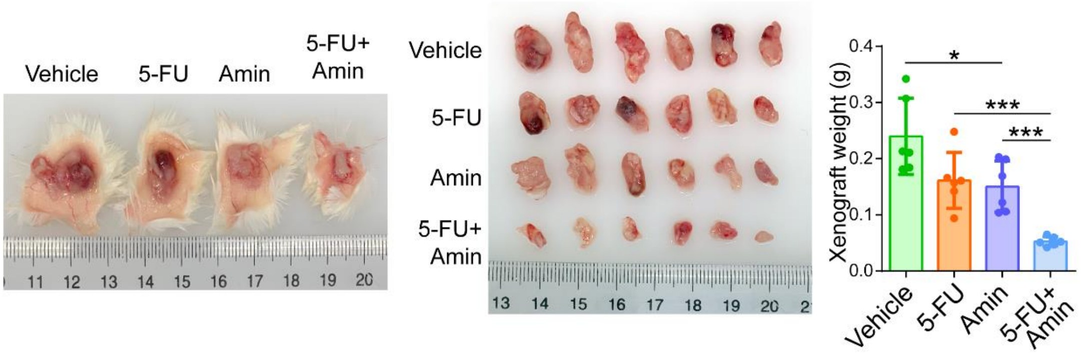

Supplementary Figure S11 Administration of aminopterin inhibits subcutaneous xenograft growth, and mice in the aminopterin-5-FU co-administration group generated smaller xenografts (n = 6/group; *, $P < 0.05$; ***, $P < 0.001$).

Supplementary Figure S12

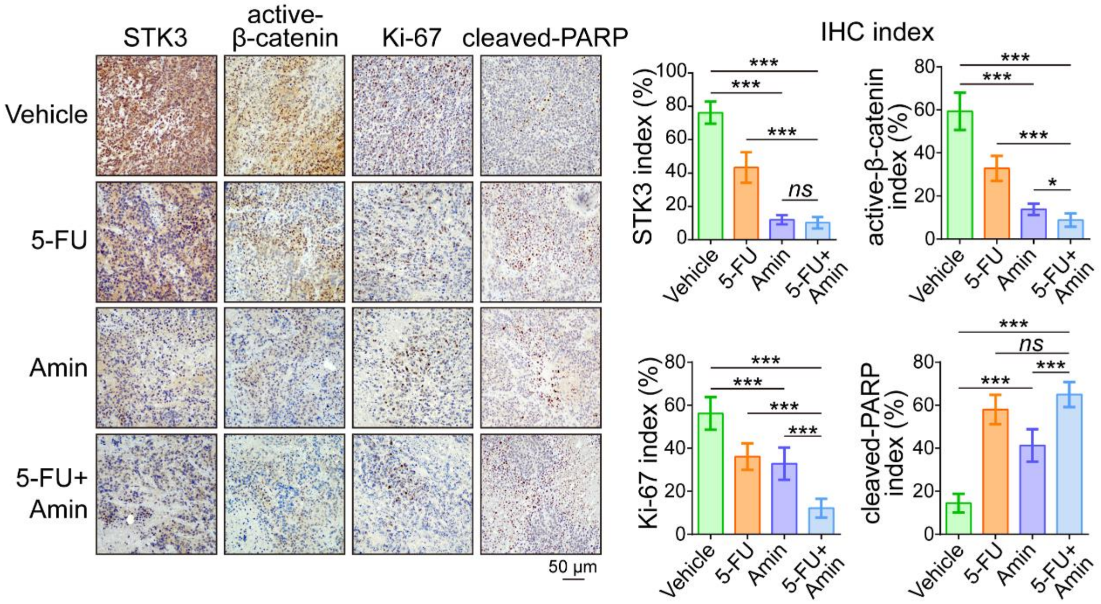

Supplementary Figure S12 IHC staining of STK3, active-β-catenin, Ki-67 and cleaved-PARP on the xenografts harvested from aminopterin sole or co-administration treatment groups (*ns*, not significant; *, $P < 0.05$; ***, $P < 0.001$).

Supplementary Figure S13

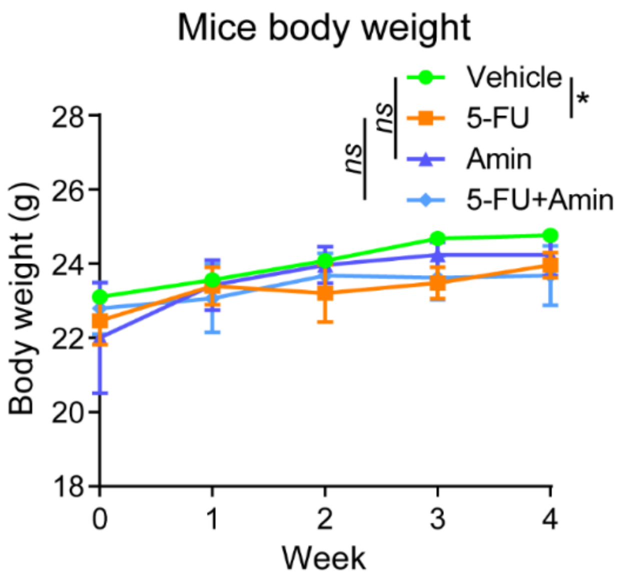

Supplementary Figure S13 Records of mice body weight during aminopterin and/or 5-FU treatment (n = 6/group; *ns*, not significant; *, $P < 0.05$).

Supplementary Figure S14

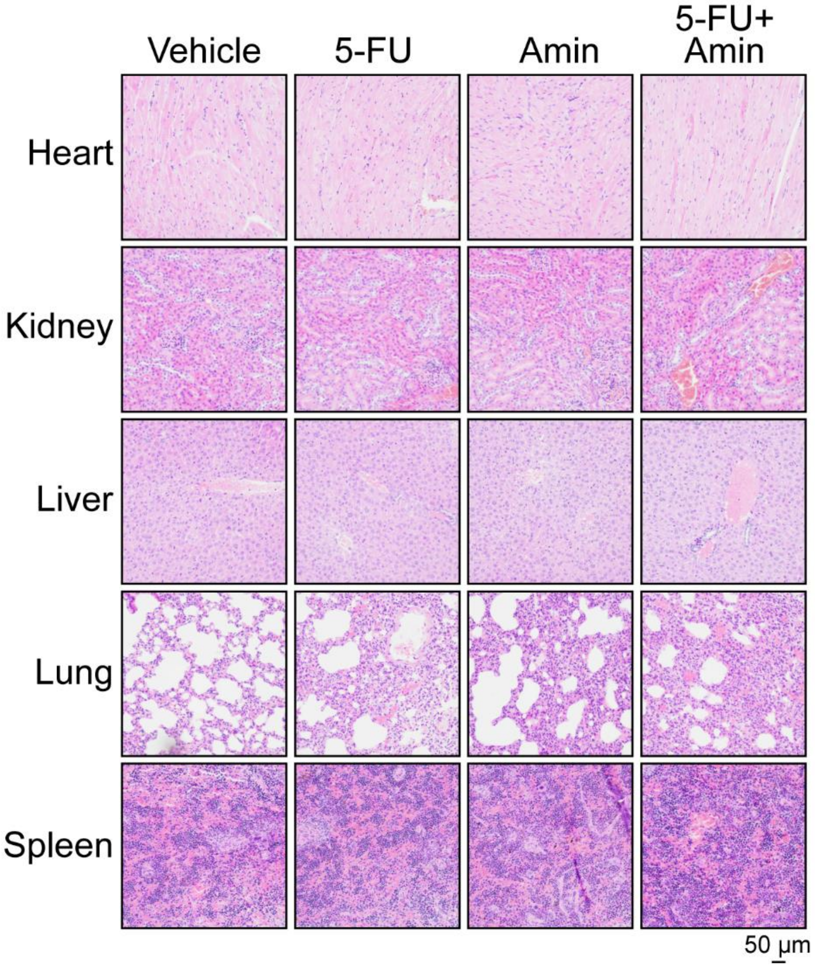

Supplementary Figure S14 Representative H&E staining images of the major organs collected from aminopterin and/or 5-FU treatment groups.
