# Supplementary material for: STK3 is a transcriptional target of YAP1 and a hub component in the crosstalk between Hippo and Wnt signaling pathways during gastric carcinogenesis
Source: Mol Cancer. 2025 Jul 2;24:186. doi: 10.1186/s12943-025-02391-x (PMC12220525; doi:10.1186/s12943-025-02391-x)

Uncropped Gels and Blots images for

STK3 is a transcriptional target of YAP1 and a hub component in the crosstalk between Hippo and Wnt signaling pathways during gastric carcinogenesis

Fuda Xie[1,2,3], Yang Lyu[1], Bonan Chen[1,2,3], Hoi Wing Leung[1], Peiyao Yu[1], Tiejun Feng[1], Canbin Fang[1], Alvin H.K. Cheung[1], Bin Zhou[4], Jianhui Jiang[5], Ge Zhang[6], Dazhi Xu[7], Liang Li[8], Chen Jiang[9], Jianwu Chen[10], Zhaocai Zhou[11], Liwei An[12], Bing Huang[13], Kangmin Zhuang[14], Xiaobei Luo[15], Kam Tong Leung[16], Ching Hei To[17], Brigette BY Ma[17], Chi Chun Wong[2], William KK Wu[18], Jun Yu[2,19], Ka Fai To[1], Wei Kang[1,2,3,§]

Figure 1B

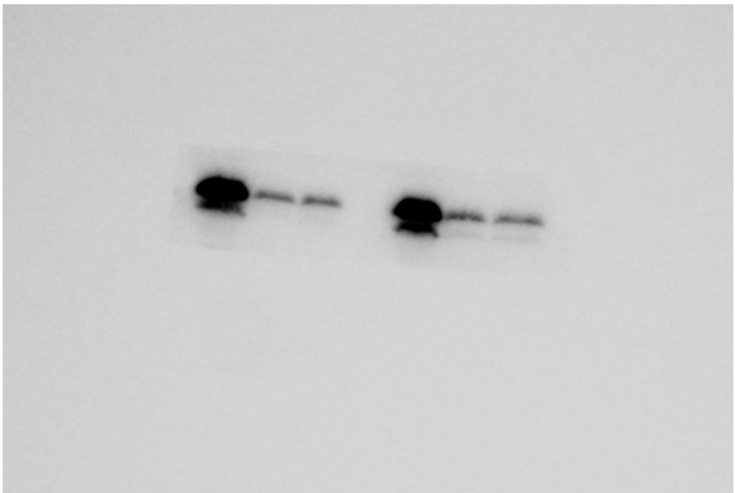

YAP1

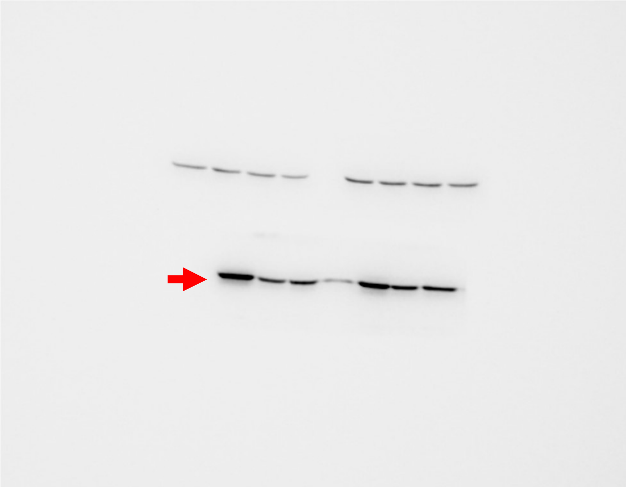

STK3

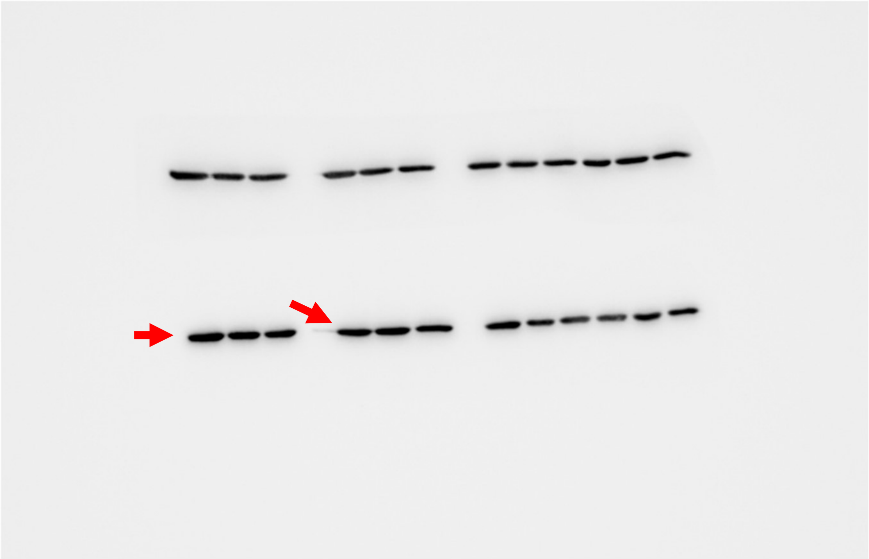

β-actin

Figure 1C

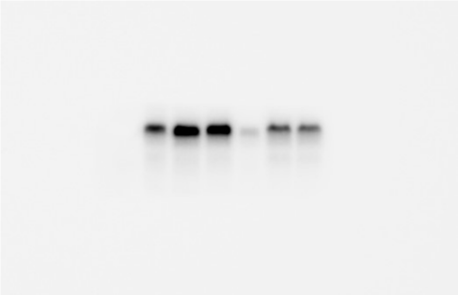

YAP1

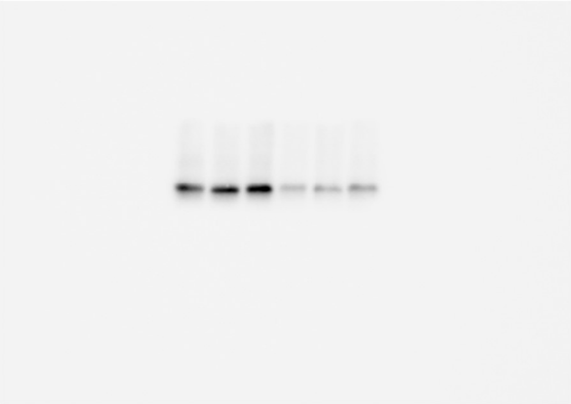

STK3

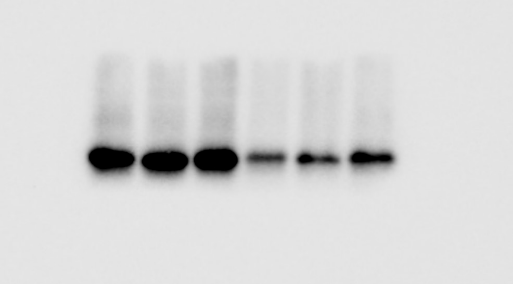

(long exposure)

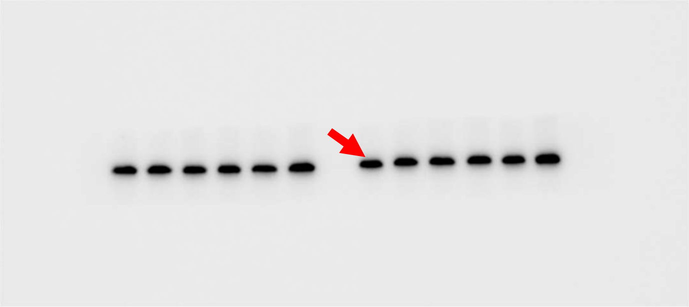

β-actin

Figure 1G

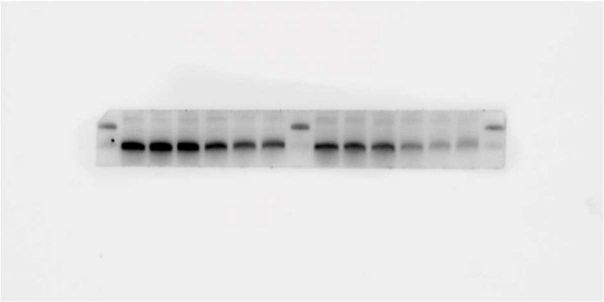

YAP1

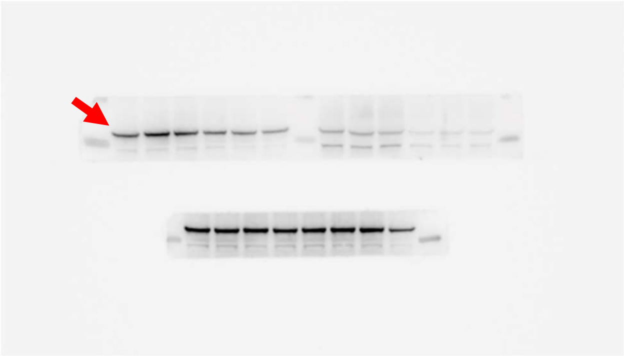

STK3 (MKN28)

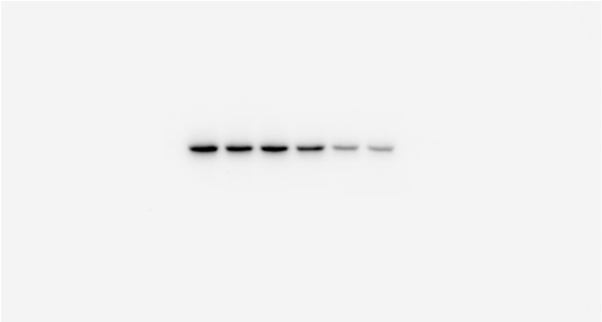

STK3 (NCI-N87)

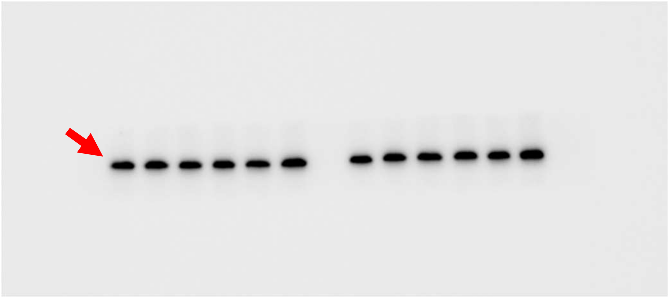

β-actin (MKN28)

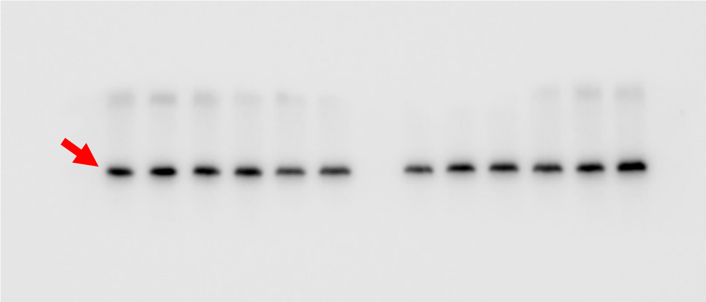

β-actin (NCI-N87)

Figure 1H

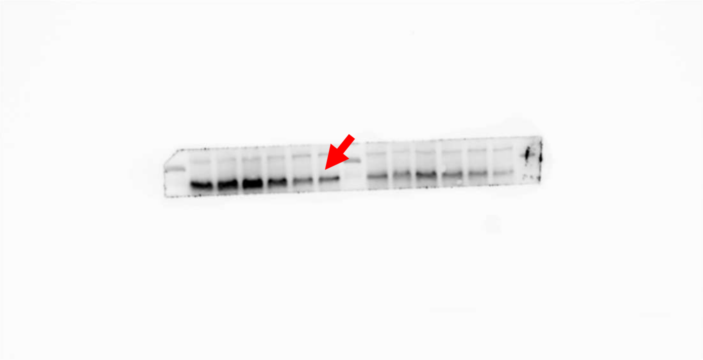

YAP1 (MKN28)

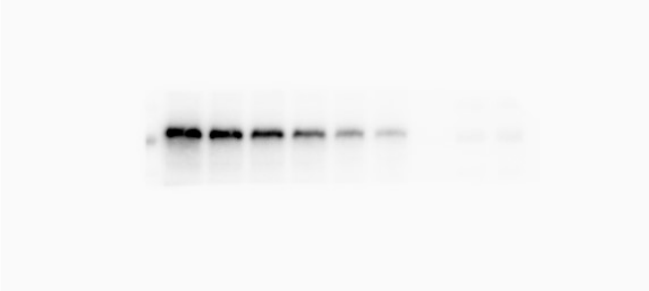

YAP1 (NCI-N87)

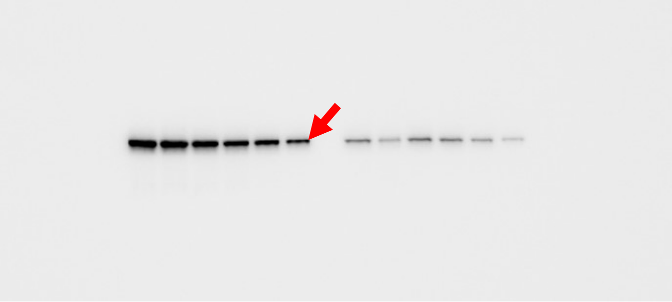

STK3 (MKN28)

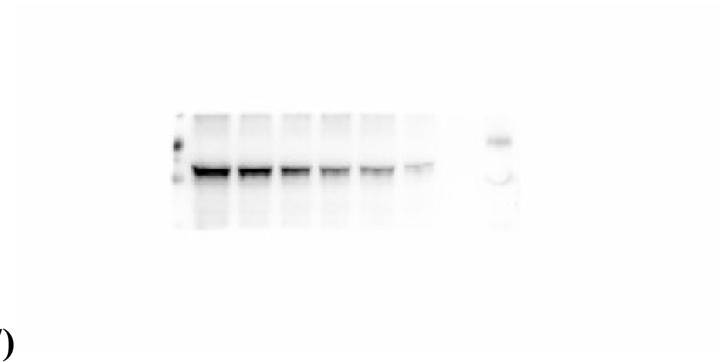

STK3 (NCI-N87)

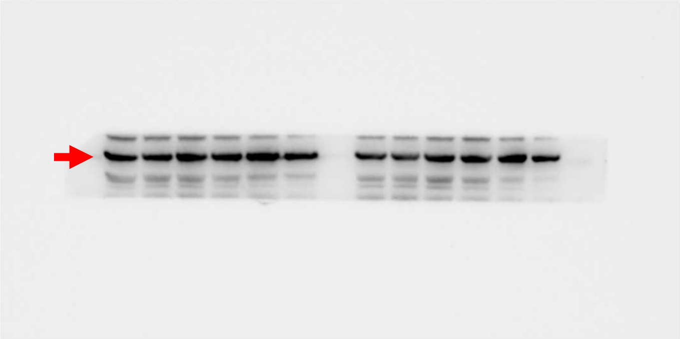

β-actin

Figure 3N

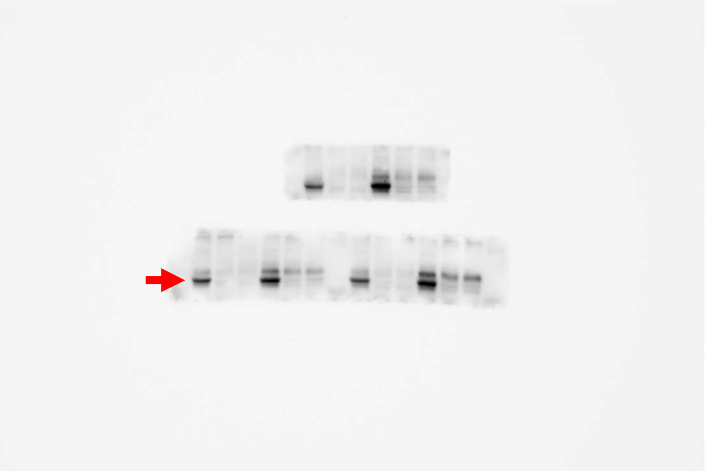

STK3

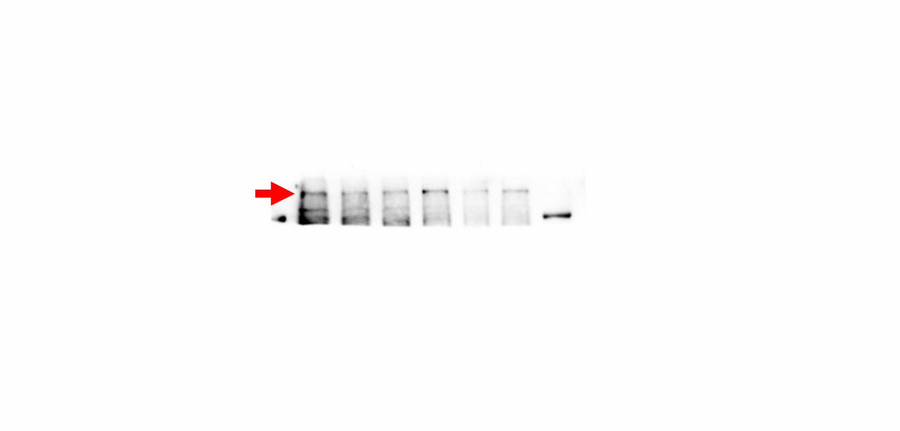

pATM

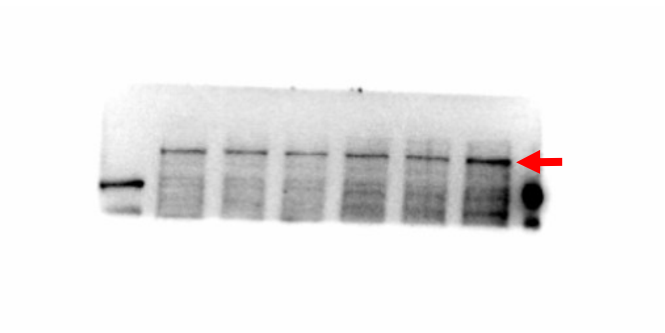

ATM

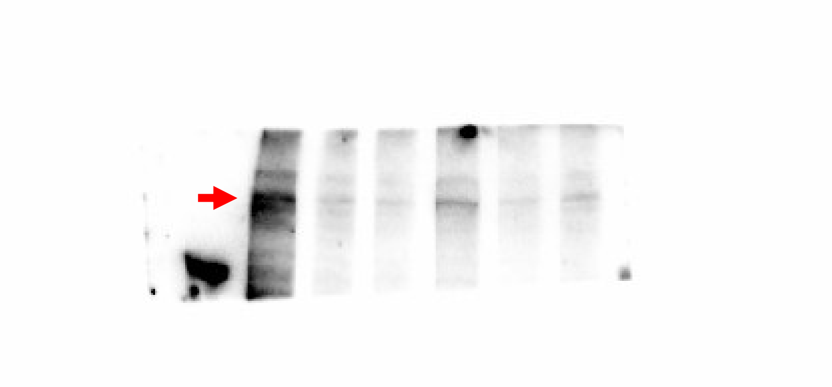

pATR

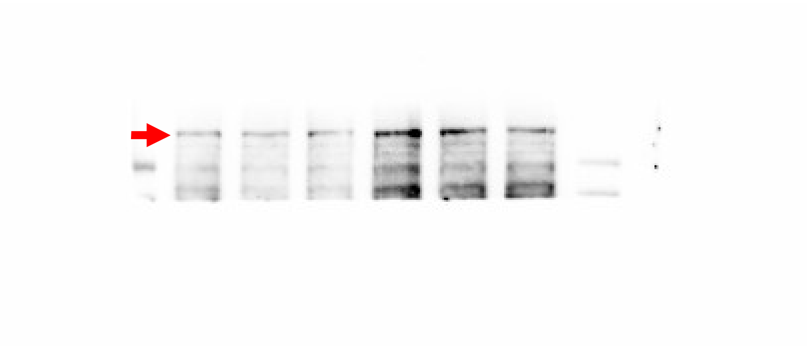

ATR

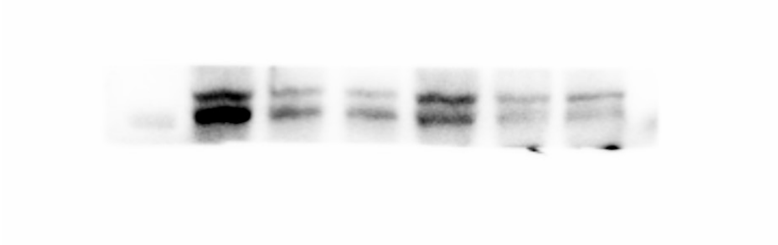

pCHK1

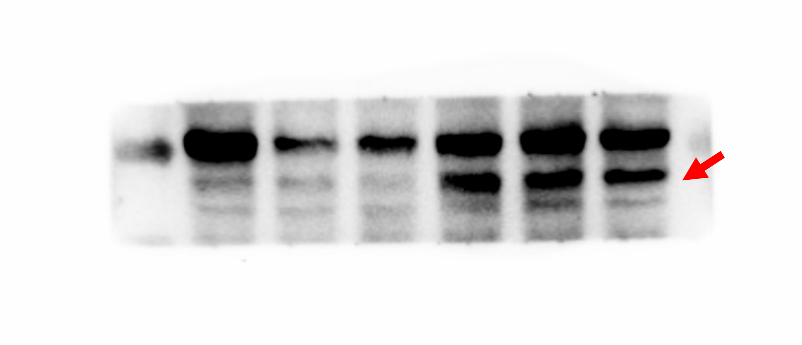

CHK1

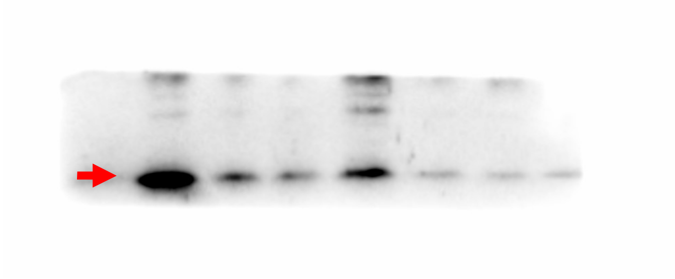

α-H2AX

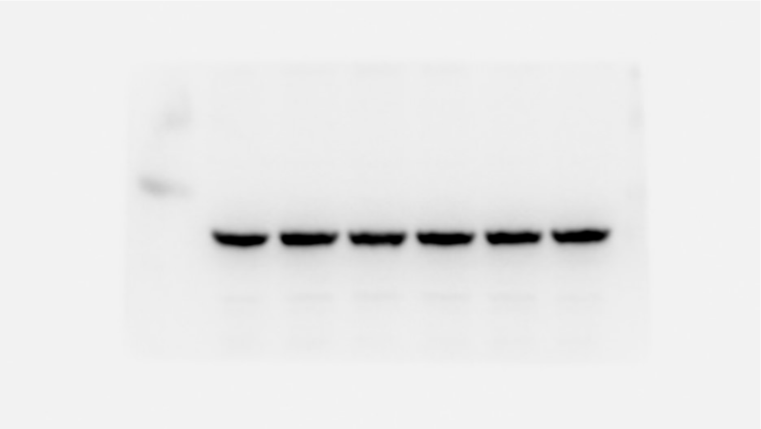

β-actin

Figure 4E

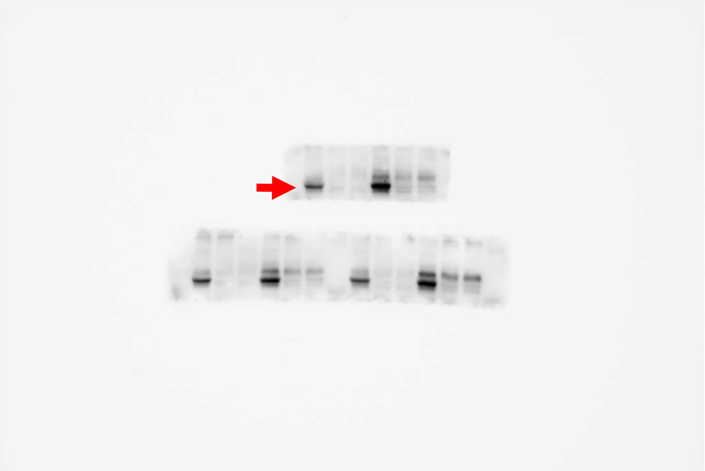

STK3

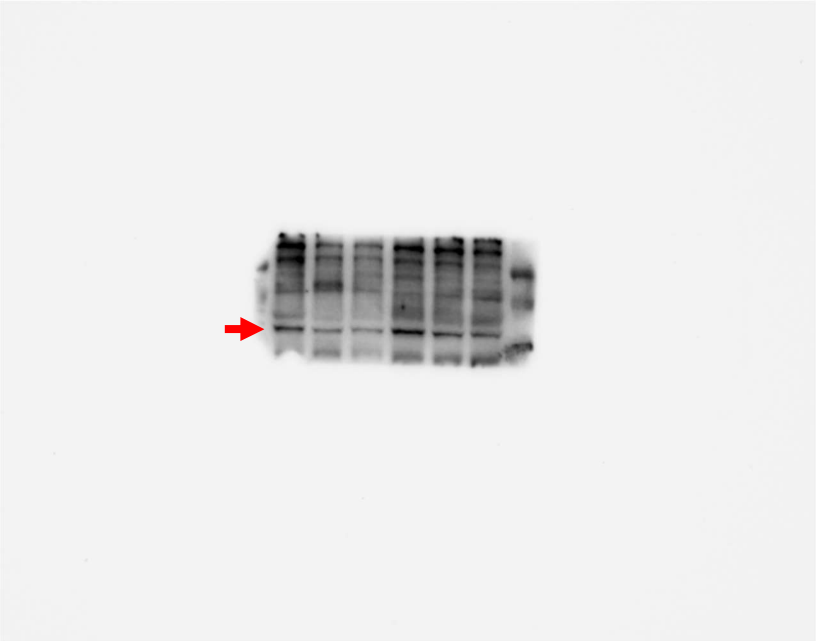

CD44

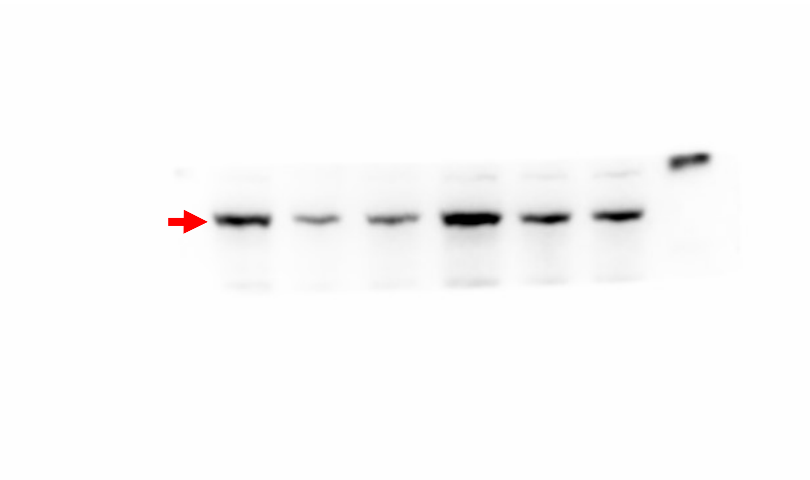

Nanog

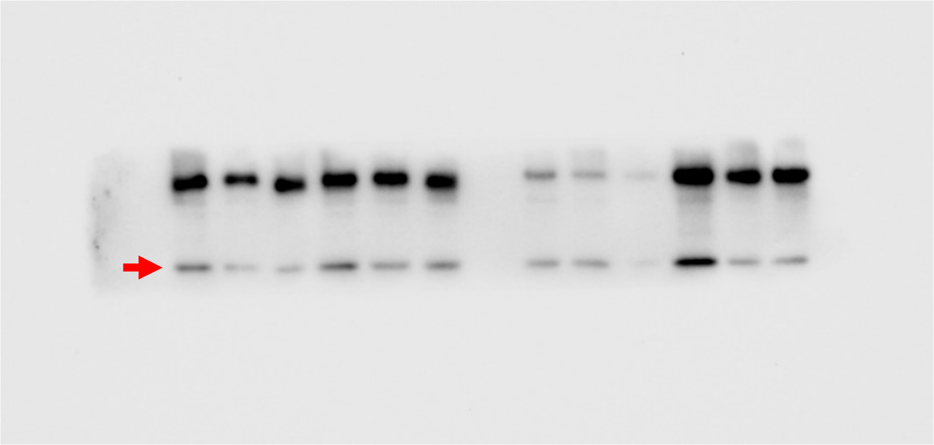

KLF4

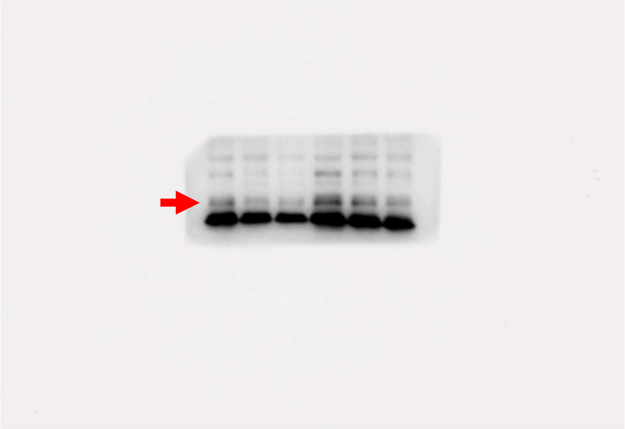

SOX2

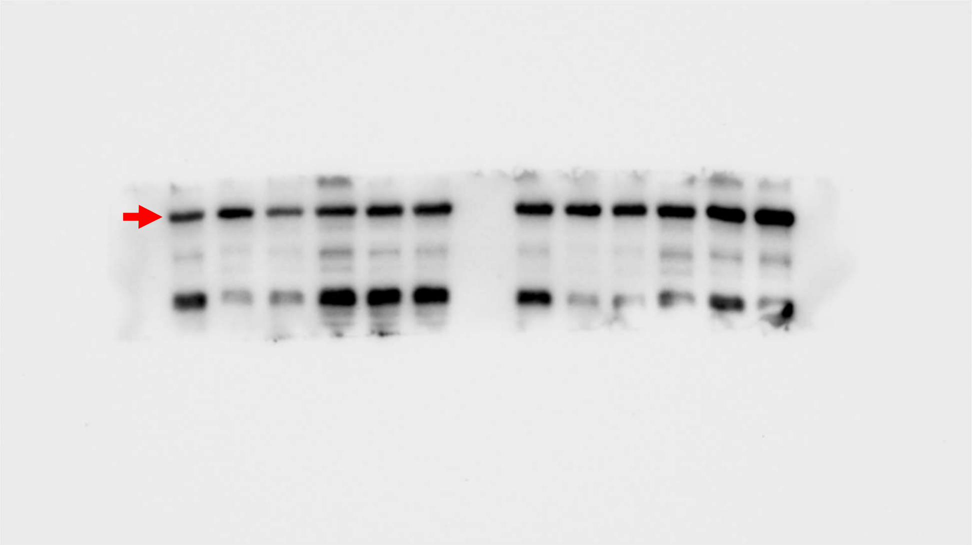

β-actin

Figure 4F

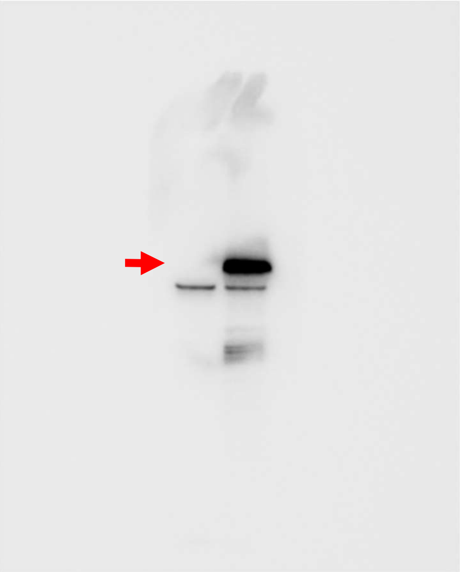

STK3

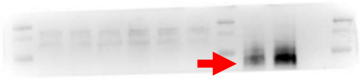

CD44

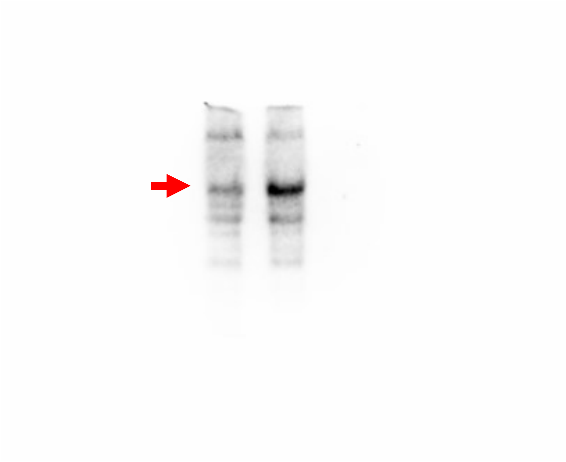

Nanog

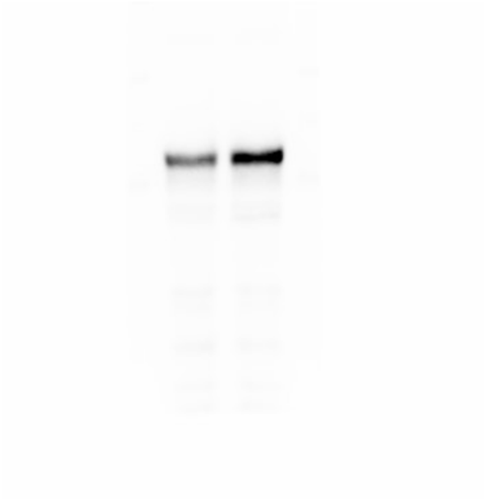

KLF4

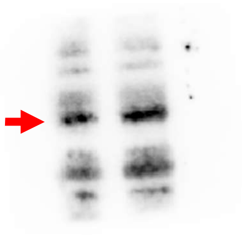

SOX2

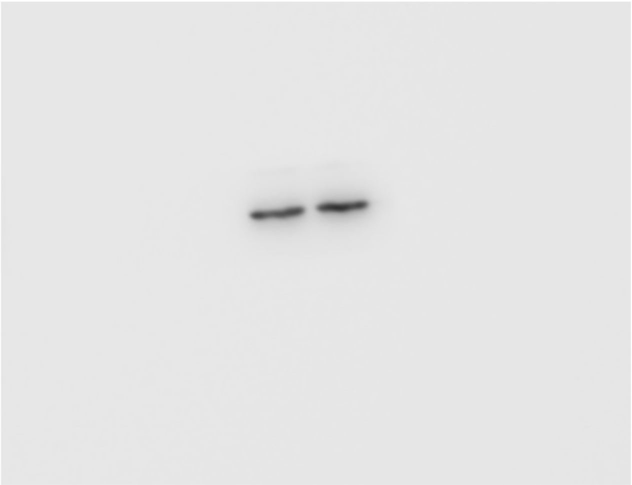

β-actin

Figure 6G

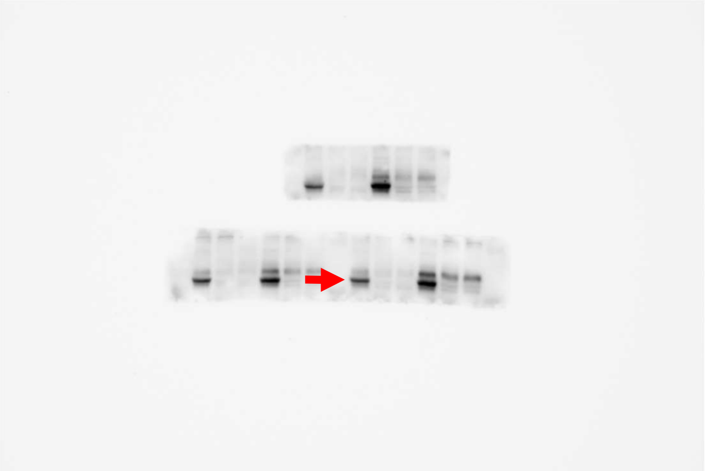

STK3

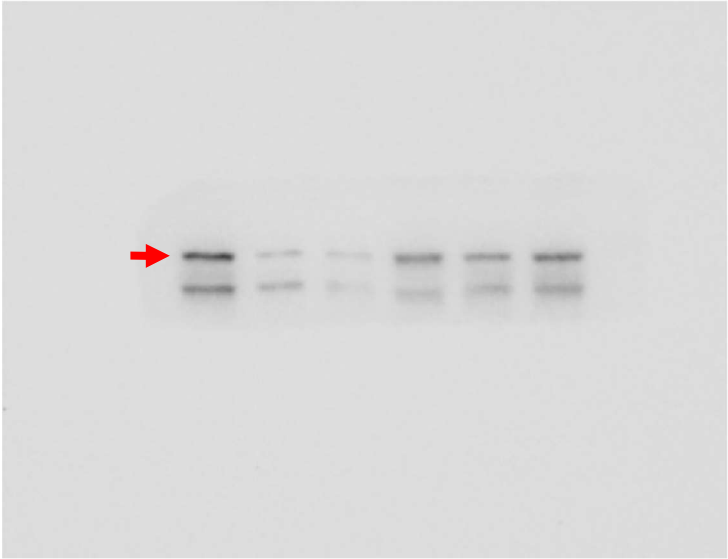

phospho-GSK3β

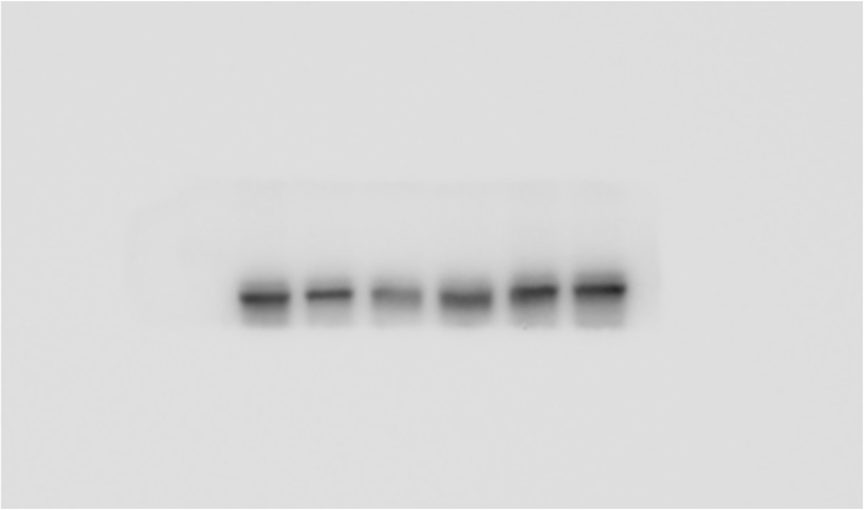

GSK3β

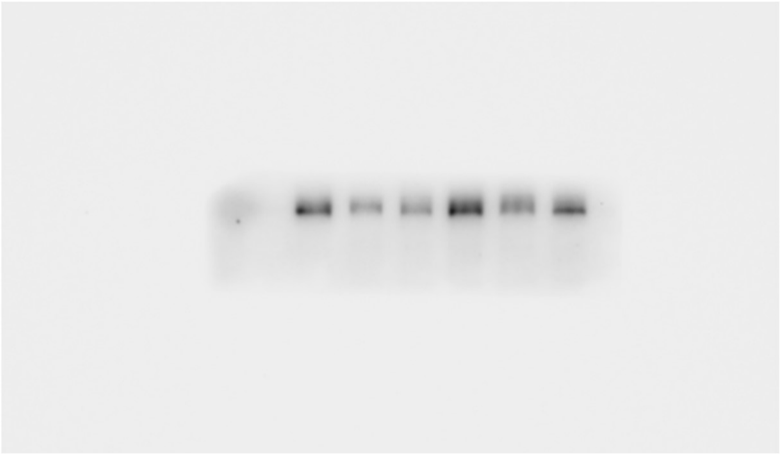

active β-catenin

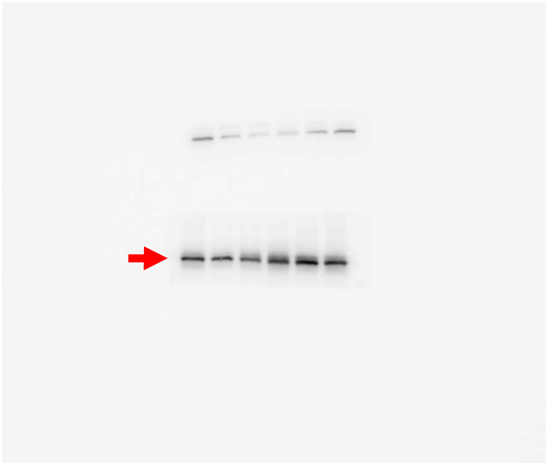

total β-catenin

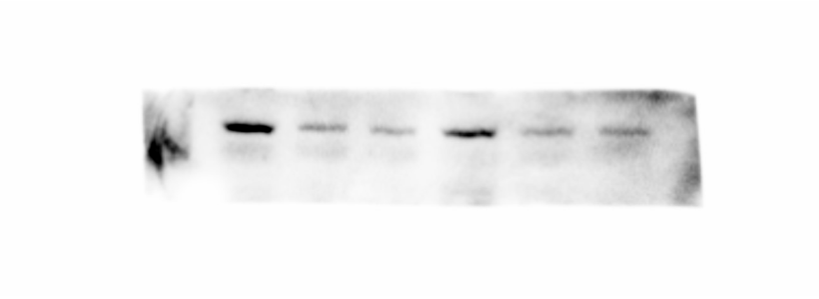

c-Myc

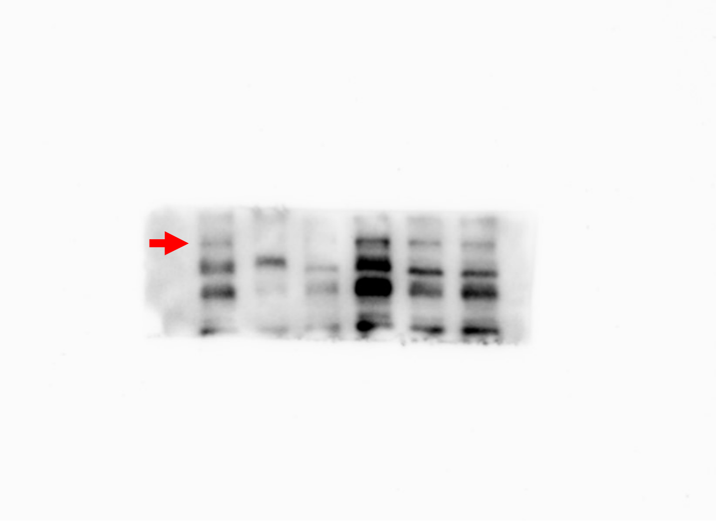

Cyclin D1

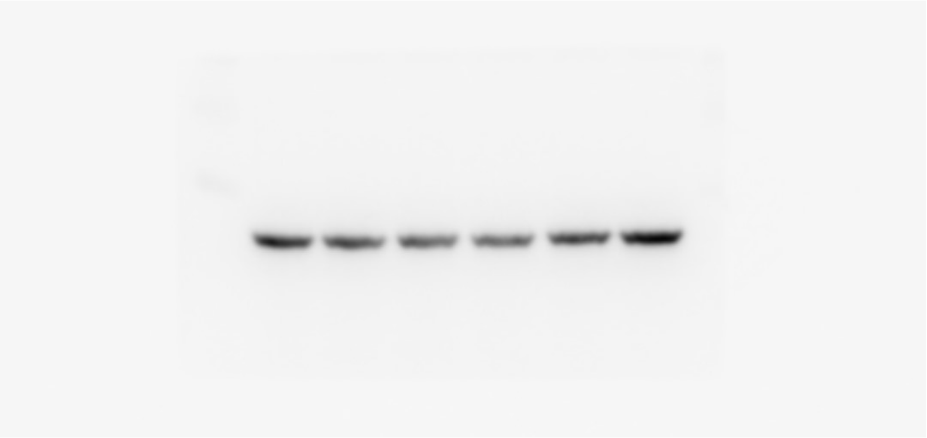

β-actin

Figure 6H

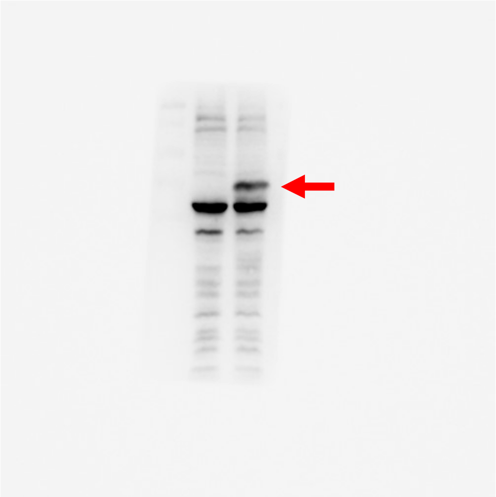

STK3

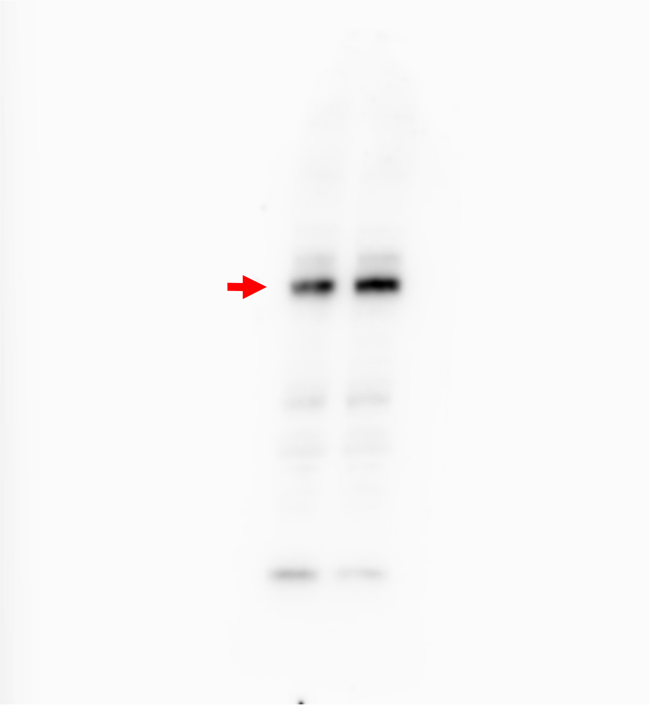

phospho-GSK3β

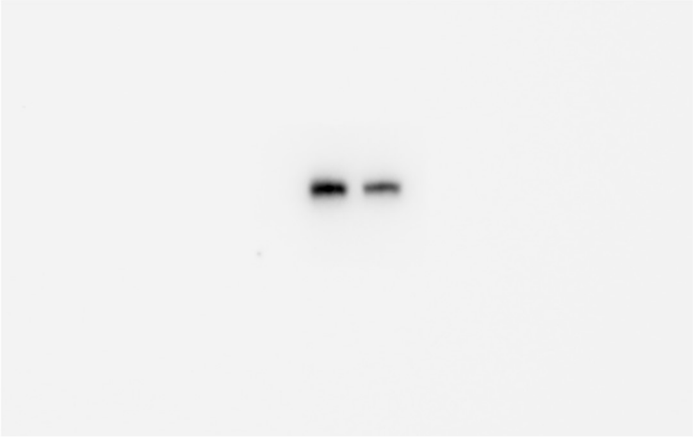

GSK3β

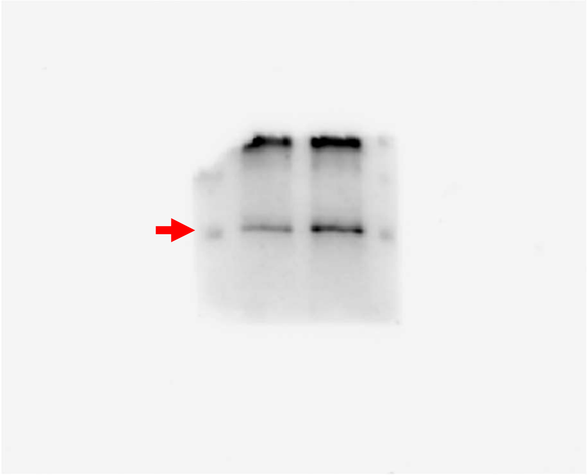

active β-catenin

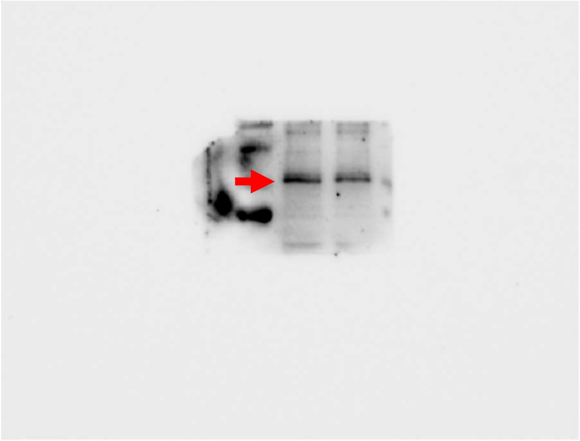

total β-catenin

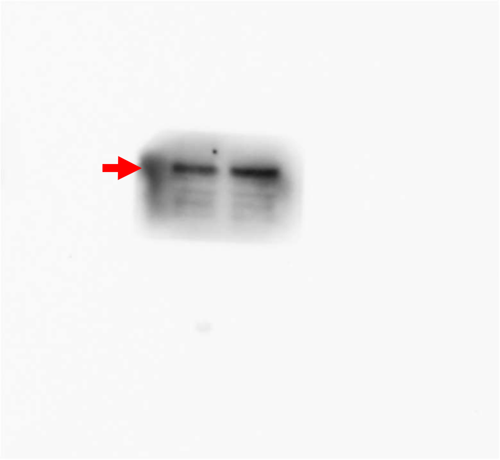

c-Myc

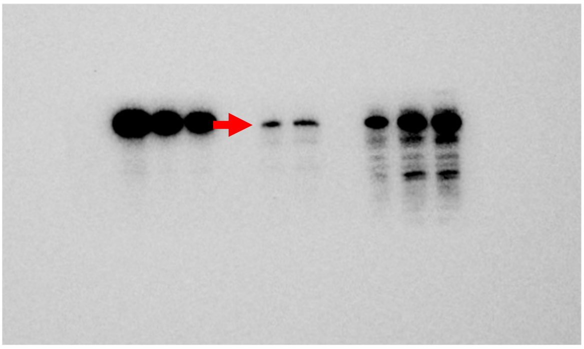

Cyclin D1

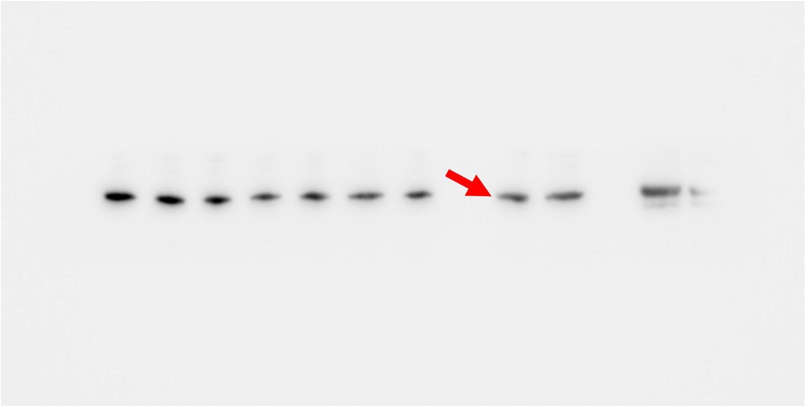

β-actin

Figure 6J

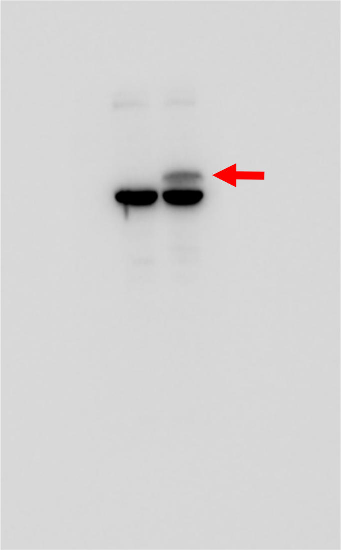

Flag-tag (IP)

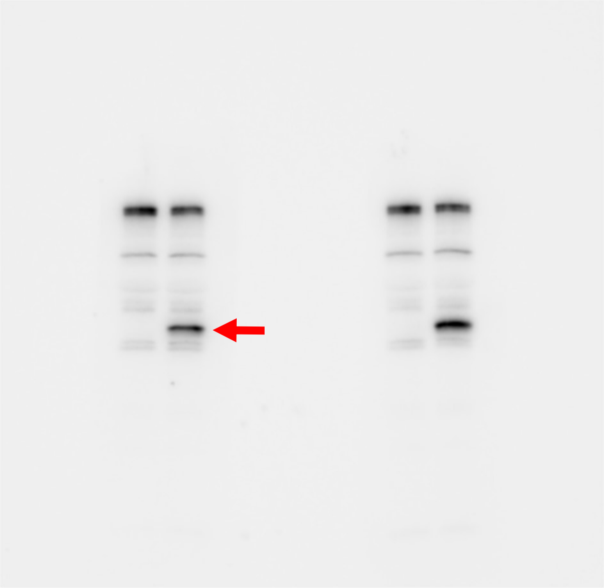

HA-tag (IP)

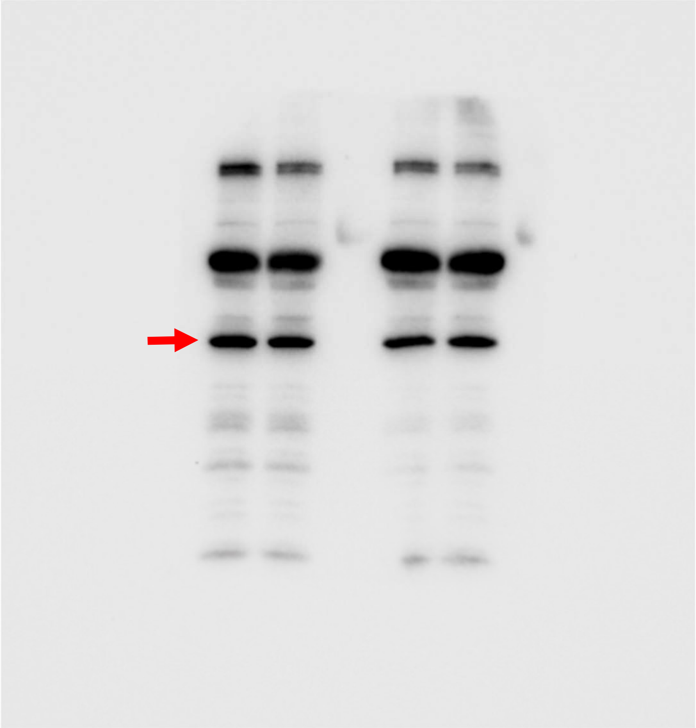

Flag-tag (Input)

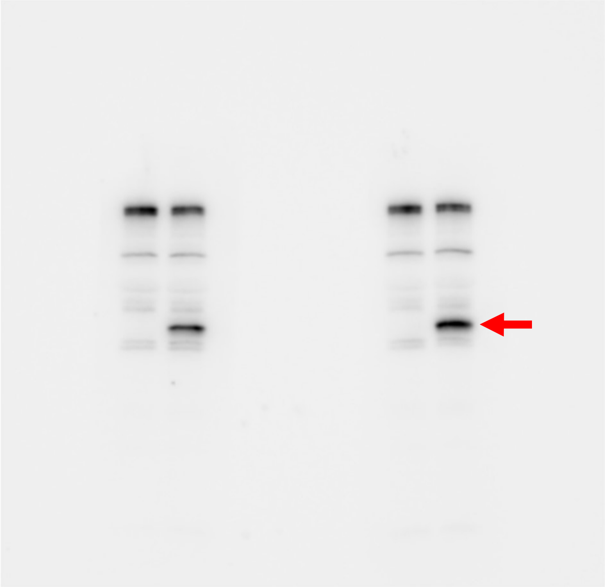

HA-tag (Input)

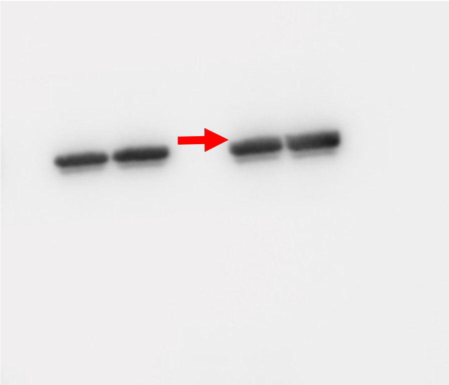

β-actin (Input)

Figure 6K

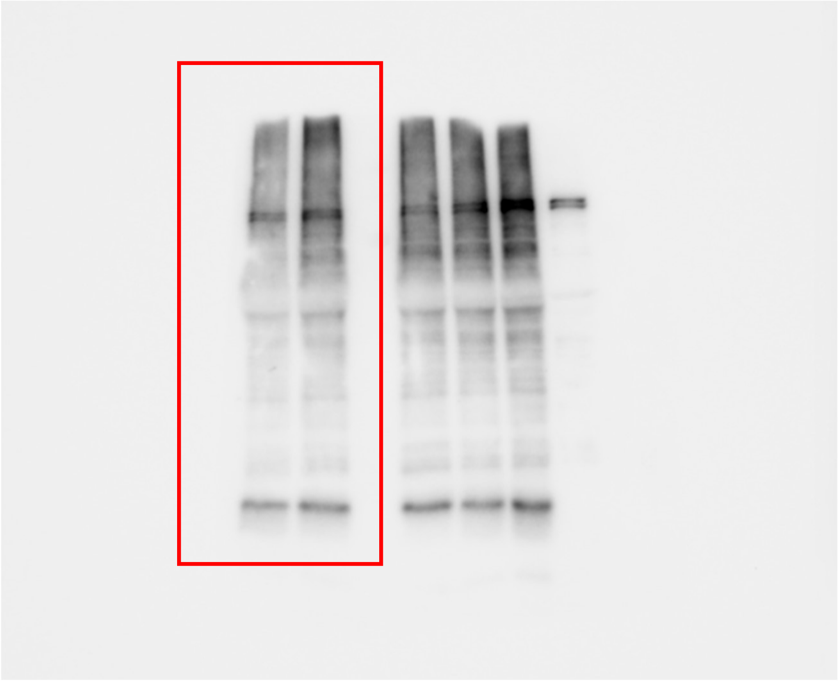

HA-tag (IP)

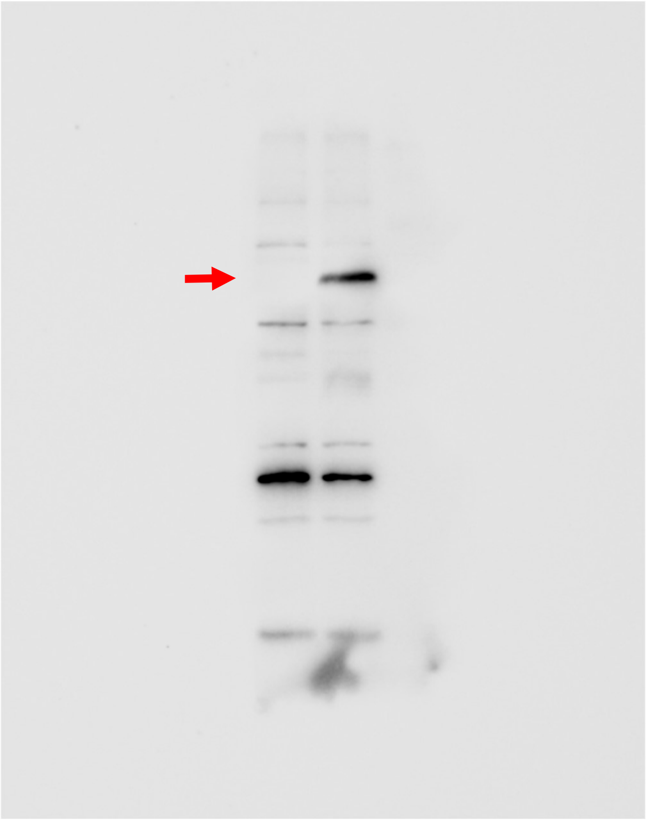

Flag-tag (Input)

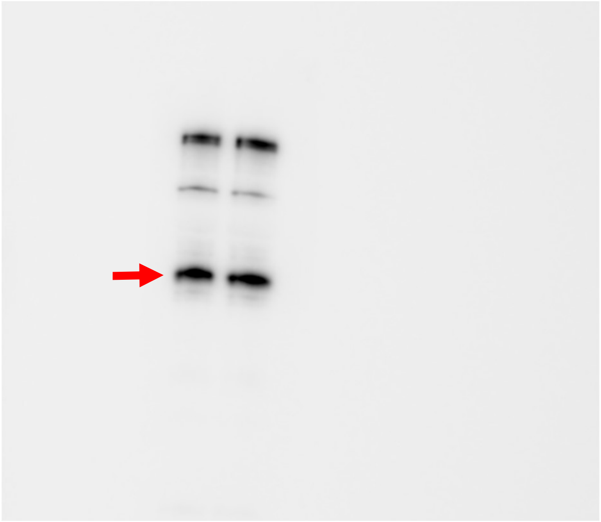

HA-tag (Input)

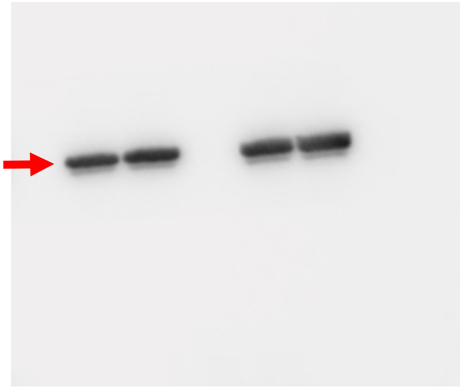

β-actin (Input)

Figure 7E

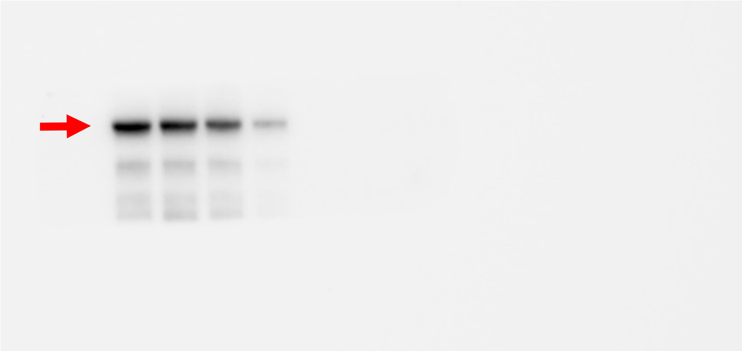

STK3 (DMSO)

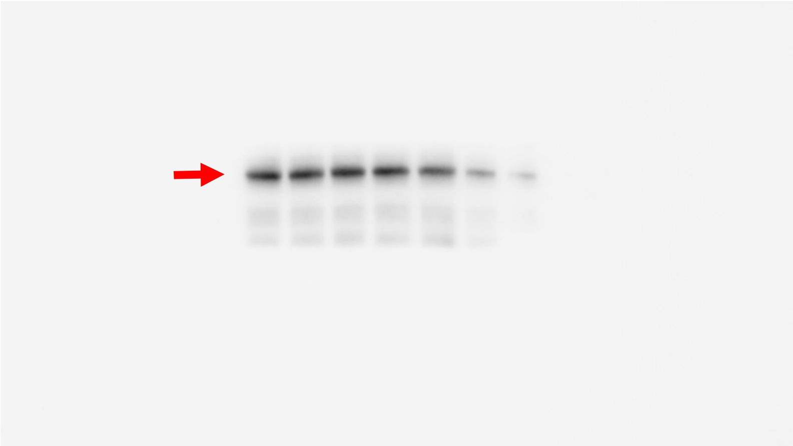

STK3 (Amin)

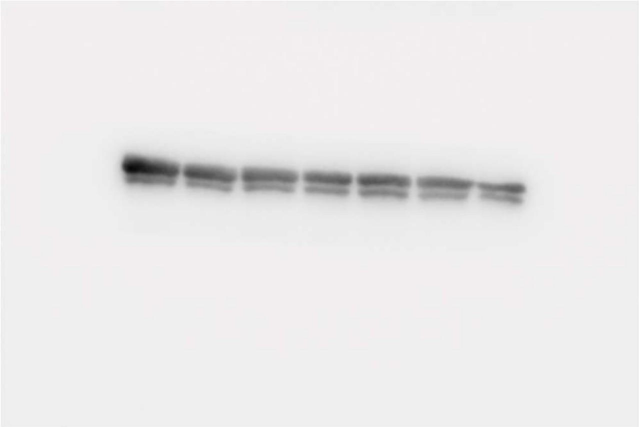

β-actin (DMSO)

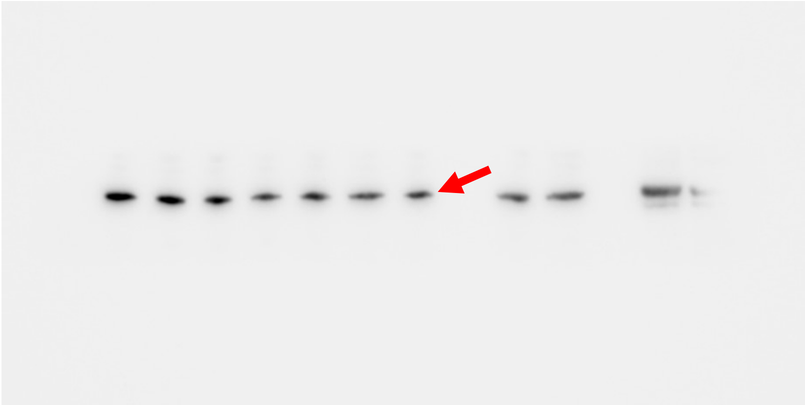

β-actin (Amin)

Figure 7H

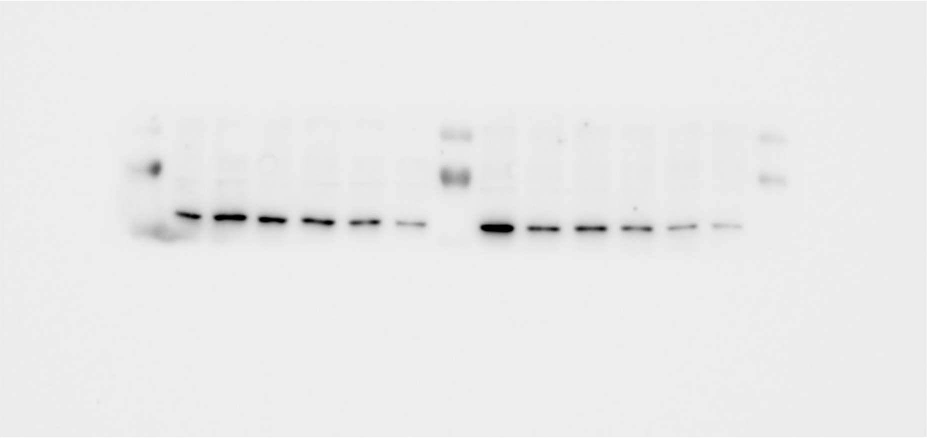

STK3

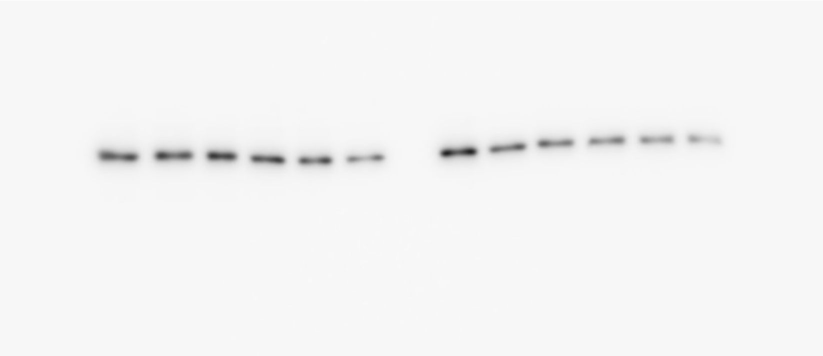

pGSK-3β (S9)

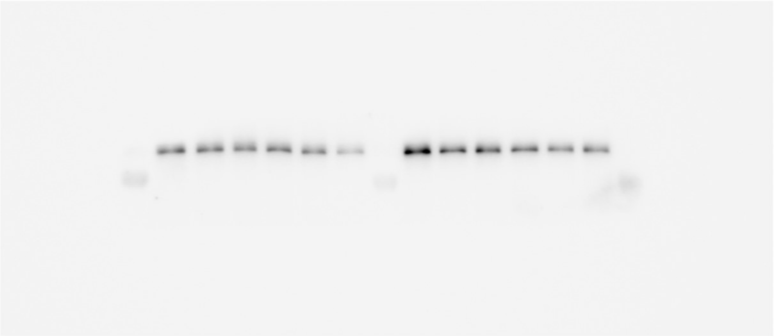

ABC

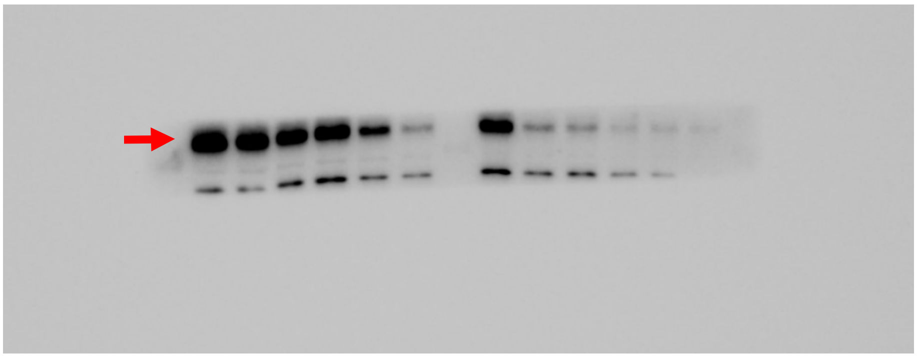

c-Myc

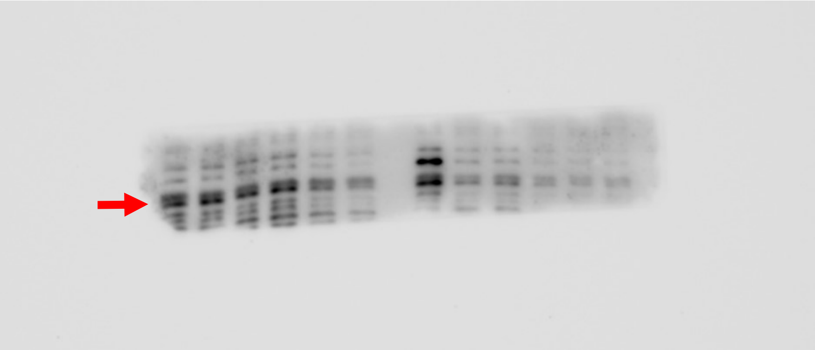

Cyclin D1

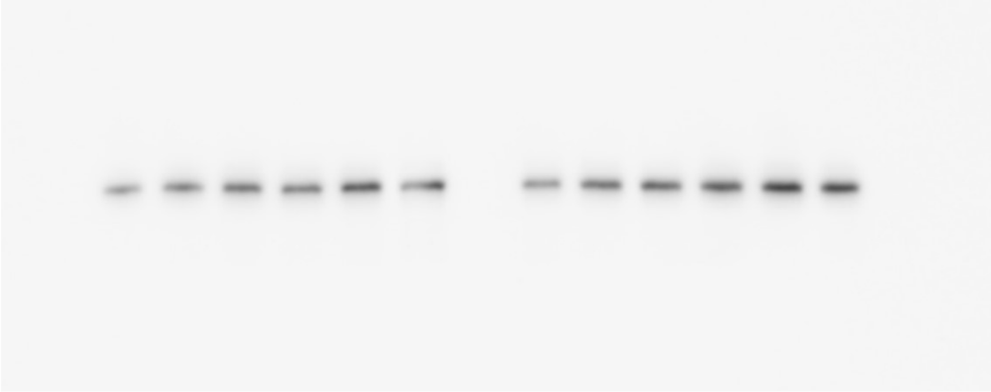

p27

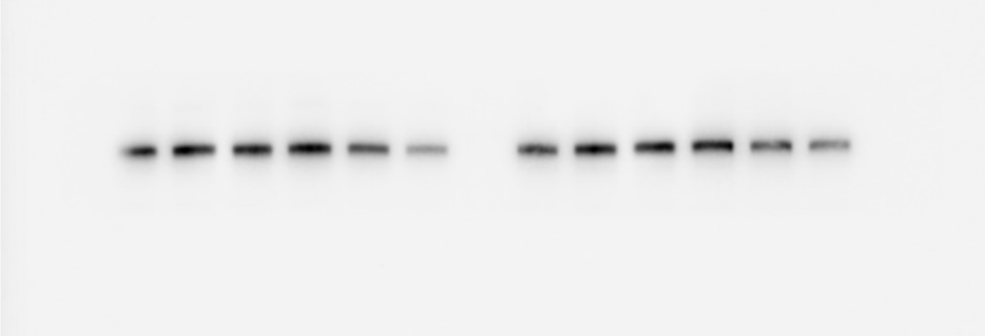

pRb

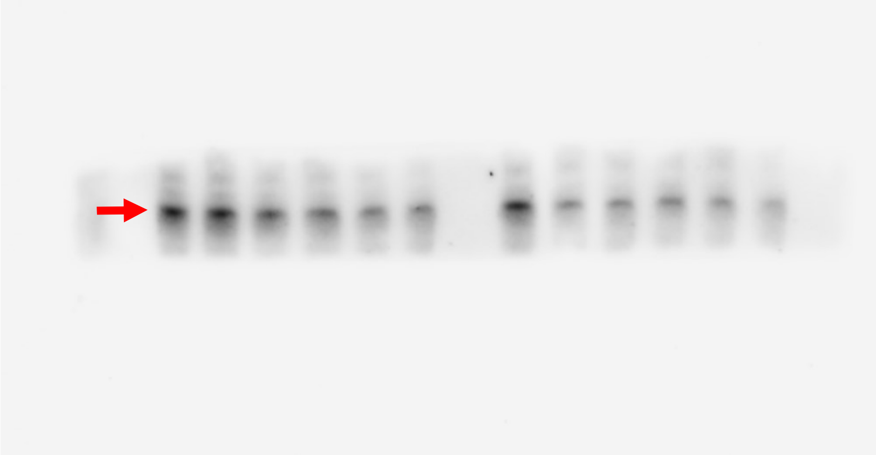

CDK4

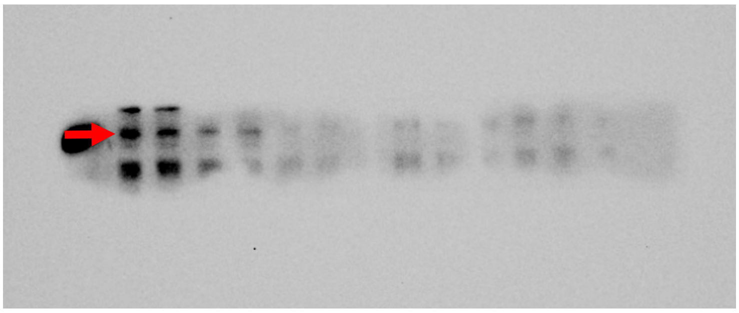

CDK6 (NCI-N87)

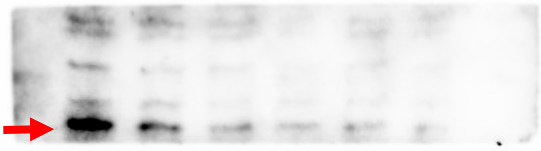

CDK6 (MKN28)

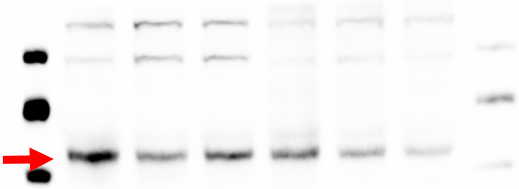

CD44 (NCI-N87)

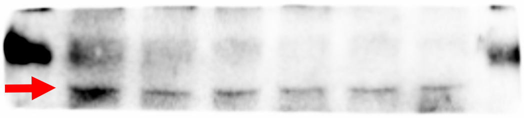

CD44 (MKN28)

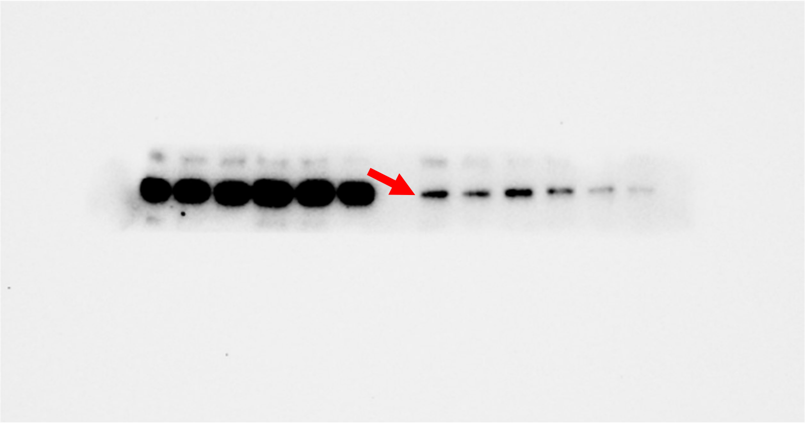

Nanog (NCI-N87)

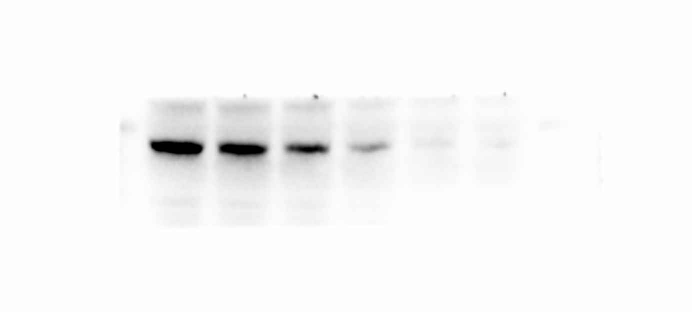

Nanog (MKN28)

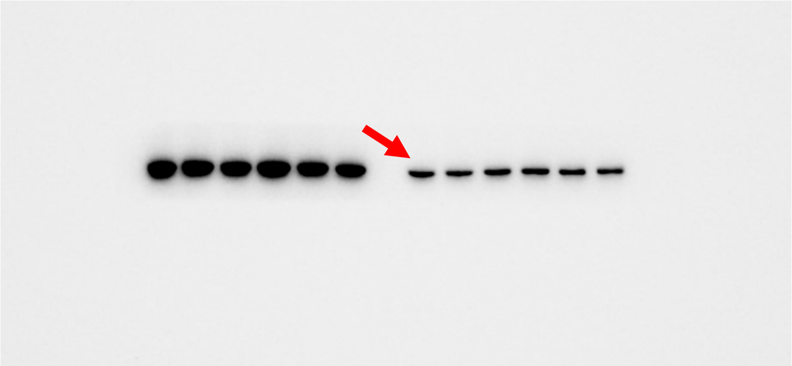

β-actin (NCI-N87)

β-actin (MKN28, short exposure)

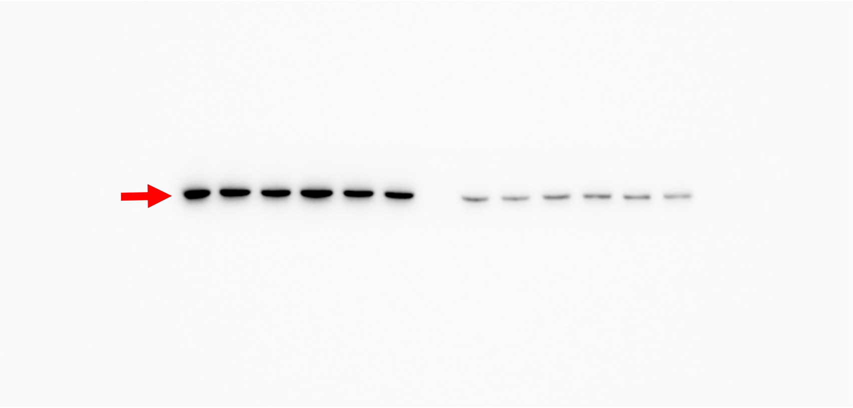

Supplementary Figure 2

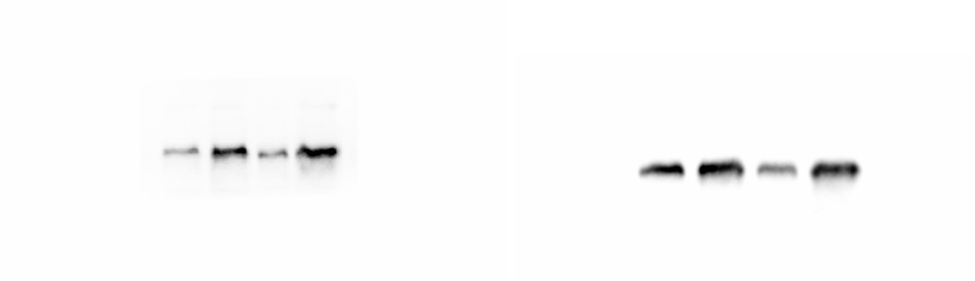

YAP1

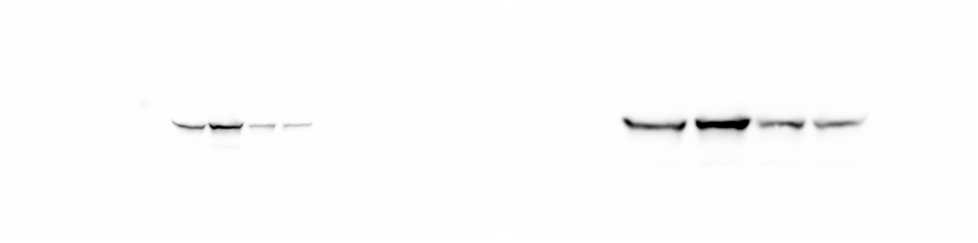

STK3

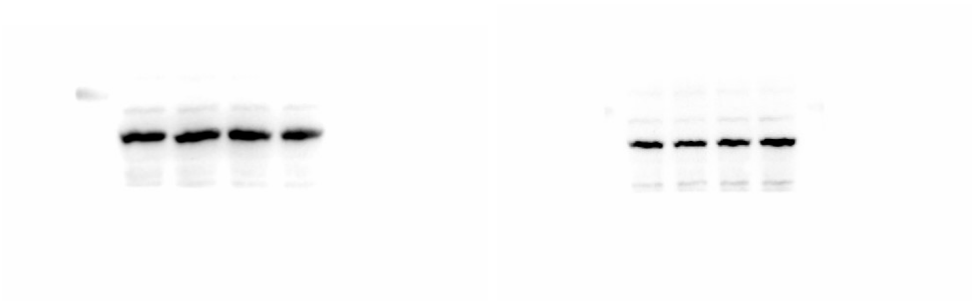

β-actin

Supplementary Figure 3

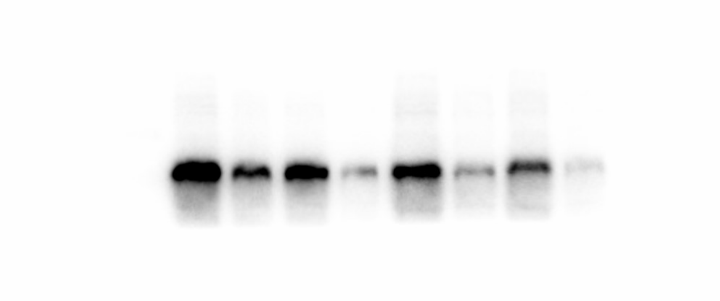

YAP1

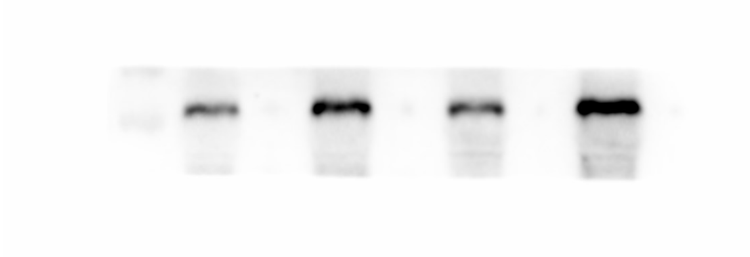

p-YAP1

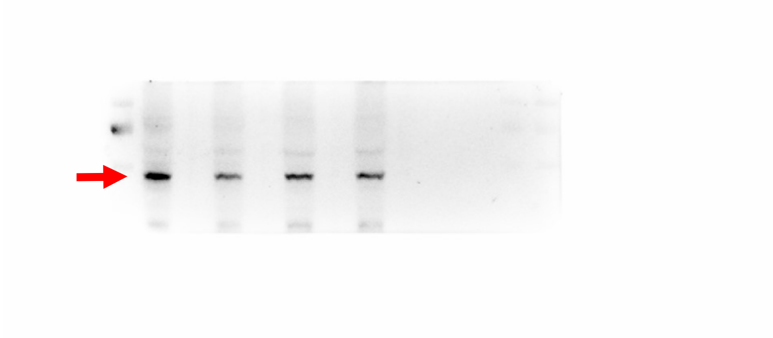

α-Tubulin

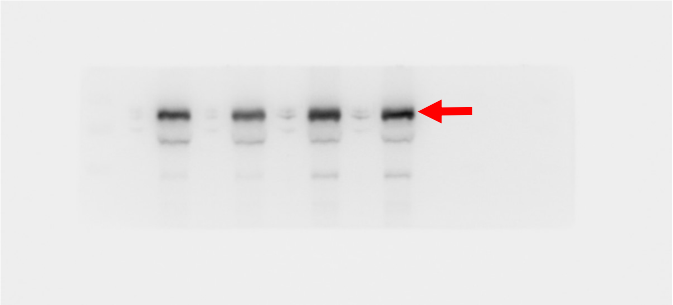

Lamin A

Supplementary Figure 4

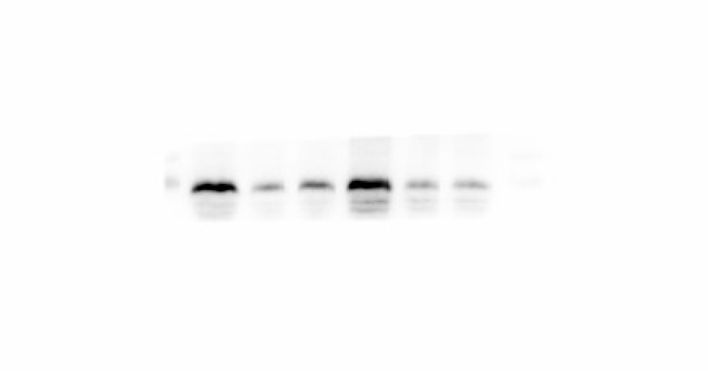

STK3

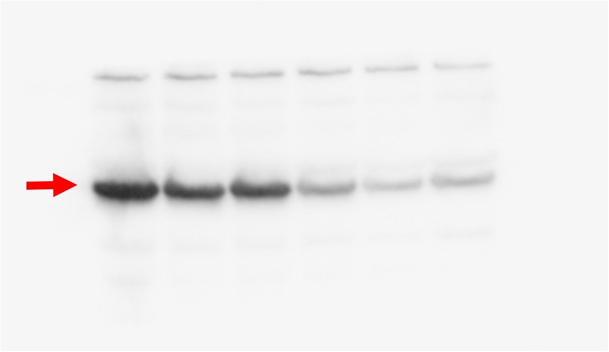

YAP1

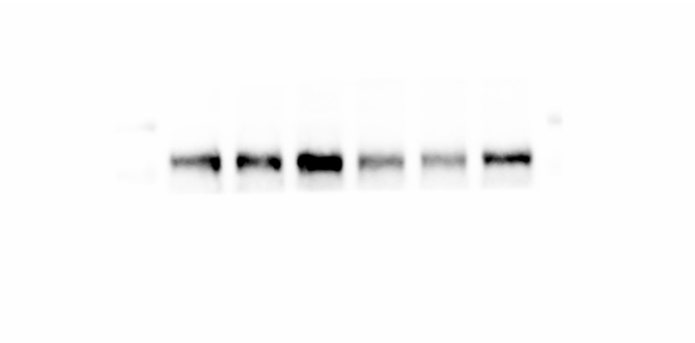

p-YAP1

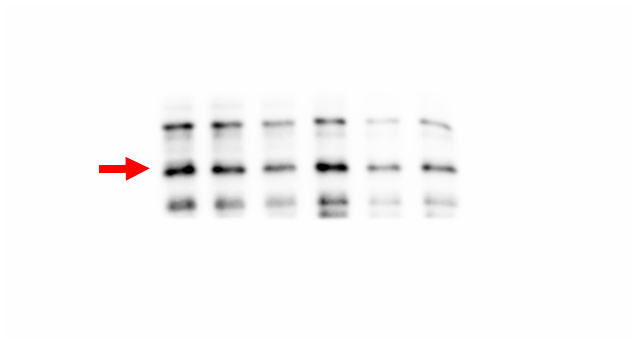

CTGF

β-actin

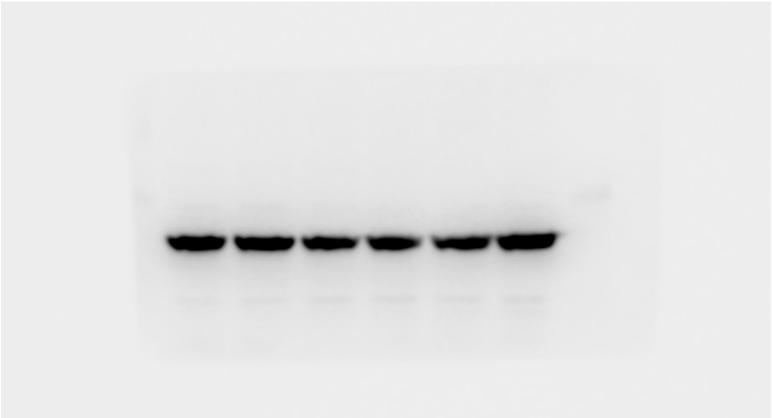

Supplement: Supplementary file 3 — Supplementary Material 3 [file 12943_2025_2391_MOESM3_ESM.pdf]
